# Optimal control of spiral waves in heterogeneous excitable media: non-monotonic efficacy of localized optogenetic inhibition

**DOI:** 10.1098/rsif.2025.1341

**Published:** 2026-09-09

**Authors:** Heqing Zhan, Kaiqi Liu, Yaolei Ju, Chuan'an Wei, Dongdong Deng, Yuanbo Yu

**Affiliations:** ^1^ School of Intelligent Medicine and Technology (Big Data Research Center), Hainan Medical University, Haikou, Hainan, People's Republic of China; ^2^ School of Biomedical Engineering, Dalian University of Technology, Dalian, People's Republic of China

**Keywords:** excitable media, spiral wave, optogenetic control, structural heterogeneity, non-monotonic response, wave annihilation, pattern formation, spatio-temporal dynamics

## Abstract

Structural heterogeneity stabilizes spiral waves in excitable media, yet how its spatial architecture, ranging from diffuse interstitial deposition to focal scar patterns, modulates the efficacy of localized optogenetic control remains poorly understood. Using a two-dimensional computational model of human atrial tissue expressing the inhibitory opsin GtACR1, we systematically evaluated spiral wave termination across fibrosis coverage from 0 to 100%, two distinct spatial patterns, and various illumination strategies, including varied coverage, intensity profiles and low-intensity periodic stimulation. We identify a robust non-monotonic dependence of termination efficacy on illuminated area, with distinct dynamical origins for diffuse and focal fibrosis. Diffuse heterogeneity promotes efficient termination via drift toward tissue boundaries, while focal fibrosis strongly anchors the spiral core and requires substantially broader illumination for effective suppression. An optimal window covering 40–70% of the domain maximizes annihilation efficiency by reshaping the functional excitable domain and shortening the spiral core's drift path to the boundary. Excessive sub-global coverage paradoxically prolongs persistence via narrow-strip geometric confinement. Wavefront fragment collisions in focal fibrosis make only a minor, non-essential contribution to this non-monotonic response. This optimal window remains robust across varied biophysical, conduction and structural parameters, revealing a general principle for low-energy pattern control in disordered excitable media.

## 1. Introduction

Spiral waves are fundamental spatio-temporal patterns in excitable media, and their self-sustained rotation underlies persistent arrhythmias such as atrial fibrillation (AF), which is the most common serious cardiac rhythm disorder [1,2]. In the human atrium, these re-entrant waves are often stabilized by structural remodelling, particularly atrial fibrosis, which alters conduction properties and promotes wave anchoring [3]. Understanding how the spatial architecture of such heterogeneity influences the controllability of spiral waves is, therefore, crucial not only for arrhythmia management, but also for uncovering general principles of pattern dynamics in disordered active media [4,5].

Traditional electrical defibrillation, while effective, relies on high-energy shocks that cause pain and tissue damage, limiting its applicability [6]. This has spurred interest in low-energy alternatives, among which optogenetics has emerged as a transformative approach for precise modulation of cardiac excitability [7,8]. By expressing light-sensitive ion channels such as the inhibitory opsin GtACR1 in cardiomyocytes, subthreshold optical stimulation can terminate arrhythmias with minimal energy, offering a potential pathway toward gentler rhythm control [9–11]. However, the success of such interventions probably depends on the underlying tissue substrate—a factor often overlooked in idealized models [12].

Computational modelling provides a powerful platform to dissect these interactions under controlled conditions [13–15]. It allows systematic variation of fibrosis distribution, illumination geometry and stimulation protocols, and thus, yields mechanistic insights that are not readily accessible through experimental work alone [16,17]. Recent work has demonstrated that local excitatory optogenetic stimulation can achieve phase-independent unpinning of spiral waves anchored to anatomical obstacles, offering a dynamical basis for localized arrhythmia control [18]. However, most existing optogenetic cardiac models assume homogeneous tissue or simplified obstacles, paying insufficient attention to the clinically relevant spectrum of fibrosis patterns, from focal scars to diffuse interstitial deposition [16]. These distinct architectures exert opposing effects—focal fibrosis creates strong anchoring sites that stabilize rotors, whereas diffuse fibrosis enhances conduction heterogeneity, promoting wave fragmentation and dynamic instability [19]. Consequently, they may differentially shape the response to localized optical perturbation [20,21].

Moreover, prior studies typically employ fixed illumination parameters, neglecting nonlinear dynamical effects that arise from spatial and temporal modulation of light delivery, including wave drift, boundary-induced annihilation and the possible existence of an ‘optimal light range’, where intermediate illumination maximizes termination efficiency.

To address these gaps, we developed a two-dimensional computational model of human atrial tissue expressing GtACR1, incorporating realistic representations of fibrosis with coverage ranging from 0 to 100% in either focal or diffuse spatial patterns, along with myocyte–fibroblast coupling. We systematically evaluate spiral wave termination across illumination areas spanning 0 to 100% of the domain, four distinct spatial intensity profiles and low-intensity periodic stimulation protocols. Three core hypotheses are tested in this work. First, fibrosis type and severity jointly determine the minimal illumination area required for successful termination. Second, focal fibrosis reduces control efficacy as a result of strong rotor anchoring to the structural barrier. Third, an optimal illumination window exists, in which insufficient coverage fails to disrupt the re-entrant wave, while excessive coverage suppresses wavefront interactions and paradoxically prolongs arrhythmia persistence. To ensure the robustness of our findings, we performed extensive sensitivity analyses across key biophysical parameters, including opsin reversal potential, fibrosis-induced conduction slowing, spatial discretization and tissue anisotropy, with full results provided in the supplementary material. Our findings reveal a non-monotonic control principle rooted in nonlinear wave dynamics, with implications extending beyond cardiology to other excitable systems in which localized feedback interacts with structural disorder.

## 2. Material and methods

### 2.1. Model framework

We developed a two-dimensional computational model of human atrial tissue expressing the inhibitory opsin GtACR1 to investigate the optogenetic control of spiral waves under varying degrees and spatial patterns of fibrosis. The simulation domain consisted of a 101 × 101 square grid with isotropic spatial resolution of 0.025 cm, corresponding to a physical tissue size of approximately 2.5 × 2.5 cm^2^. No-flux (zero-flux) boundary conditions were applied at all edges to prevent wave reflection.

### 2.2. Cellular-level model

At the cellular level, the transmembrane potential *V*_m_ of a human atrial myocyte was governed by the following ordinary differential equation:

$$\begin{array}{r} \frac{\text{d}V_{m}}{\text{d}t}=-\frac{1}{C_{m}}\left(I_{m}+I_{stim}+I_{GtACR1}\right), \end{array}$$

where *C*_m_ is the myocyte membrane capacitance, *I*_m_ represents the sum of all intrinsic ionic currents based on a human atrial action potential model [22], *I*_GtACR1_ is the light-sensitive photocurrent and *I*_stim_ denotes an external electrical stimulus current used for spiral wave initiation.

To incorporate fibroblast coupling, each atrial myocyte could be connected to *n* = 4 fibroblasts via gap junctions with intercellular conductance *G*_gap_= 2.0 nS. The coupled system was described by

$$\begin{array}{r} \frac{\text{d}V_{m}}{\text{d}t}=-\frac{1}{C_{m}}\left(I_{m}\;+\;I_{stim}\;+\;I_{GtACR1}\;+\;nG_{gap}\left(V_{m}-V_{f}\right)\right) \end{array}$$

and

$$\begin{array}{r} \frac{\text{d}V_{f}}{\text{d}t}=-\frac{1}{C_{f}}\left(I_{f}\;+\;G_{gap}\left(V_{f}-V_{m}\right)\right), \end{array}$$

where *V*_f_ is the transmembrane potential of a single fibroblast, *C*_f_ is its membrane capacitance and *I*_f_ is the fibroblast's intrinsic ionic current, modelled according to established formulations [23].

### 2.3. Optogenetic current model

The photocurrent mediated by GtACR1 was modelled using a two-state kinetic framework originally proposed by Ochs *et al.* [24], which describes transitions between closed (*C*) and open (*O*) channel states. The dynamics of the open-state probability *O* are given by

$$\begin{array}{r} \frac{\text{d}O}{\text{d}t}=k_{open}\cdot \left(1-O\right)-\;k_{close}\cdot O, \end{array}$$

where *k*_open_ is the light-dependent opening rate constant, and *k*_close_ is the closing rate constant. The resulting photocurrent is

$$\begin{array}{r} I_{GtACR1}=g_{max}\;\cdot O\cdot \left(V_{m}-E_{GtACR1}\right), \end{array}$$

where *g*_max_ denoting the maximal membrane conductance of GtACR1, and *E*_GtACR1 _= −40 mV representing the reversal potential. All kinetic parameters were adopted directly from Ochs *et al.* [17,24], who calibrated them against experimental data from GtACR1-expressing cells [25].

### 2.4. Tissue-level model and fibrosis representation

Electrical wave propagation was governed by the monodomain reaction-diffusion equation [26]:

— In non-fibrotic regions,
  $$\begin{array}{r} \frac{\partial V_{m}}{\partial t}=\nabla \left(D\nabla V\right)-\frac{1}{C_{m}}\left(I_{m}+I_{GtACR1}\right). \end{array}$$
— In fibrotic regions (i.e. where myocytes are coupled to fibroblasts),
  $$\begin{array}{r} \frac{\partial V_{m}}{\partial t}=\nabla \left(D\nabla V\right)-\frac{1}{C_{m}}\left(I_{m}+I_{GtACR1}+nG_{gap}\left(V_{m}-V_{f}\right)\right). \end{array}$$

The baseline isotropic diffusion coefficient was set to *D* = 0.00014 cm^2^ ms^–1^, yielding a conduction velocity of approximately 43.9 cm s^–1^ in homogeneous tissue. This value falls within the well-accepted physiological range of conduction velocity for human atrial myocardium and is consistent with baseline parameter configurations in peer cardiac optogenetics modelling studies [27]. To account for impaired conduction in fibrotic zones, we reduced intercellular coupling based on cell-type adjacency: a 10% reduction in diffusion was applied at interfaces between pure myocytes and fibroblast-coupled myocytes, and a 30% reduction was imposed between adjacent fibroblast-coupled myocytes [28,29].

Three tissue configurations were considered:

— Non-fibrotic: all grid points occupied by pure atrial myocytes.
— Diffuse fibrosis: fibroblast-coupled myocytes randomly and uniformly distributed across the entire domain at prescribed densities (25%, 50%, 75% and 100%). All percentage values refer to the proportion of cardiomyocytes coupled to fibro blasts, rather than complete replacement of myocytes by acellular scar tissue. Even at 100% coverage, all grid points retain functional cardiomyocytes paired with fibroblasts;
— Focal fibrosis: fibroblast-coupled myocytes spatially confined within a quasi-square region centred in the domain, with local density matching the global fibrosis percentage (e.g. 50% focal fibrosis means 50% of cells in the central region are coupled).

To evaluate the influence of stochastic fibrosis patterning on simulation outcomes, we generated multiple independent random configurations for each fibrosis percentage and spatial pattern—10 independent random distributions for each of the 25, 50 and 75% diffuse fibrosis conditions; one single simulation for 100% diffuse and focal fibrosis, as the fully fibrotic domain has no stochastic spatial variation. All termination simulations were repeated across all configurations to verify the robustness of reported results.

To systematically evaluate the influence of boundary confinement on the optogenetic termination dynamics of spiral waves, we additionally constructed an enlarged computational domain of 5.0 × 5.0 cm^2^ with a 201 × 201 pixel grid. All cellular electrophysiological parameters, diffuse fibrosis generation rules, illumination stimulation protocols and statistical criteria were kept identical to the original 2.5 × 2.5 cm^2^ model, with only spatial dimensions adjusted to isolate the interference of boundary effects. All size-control experiments were performed under the condition of 50% diffuse fibrosis with 10 independent random realizations per group.

### 2.5. Simulation protocol and stimulation scheme

The simulation consists of two sequential phases. In phase I, a stable spiral wave was induced using an S1–S2 cross-field stimulation protocol. In phase II, optogenetic intervention was applied to evaluate the efficacy of spiral wave termination. Once a stable spiral wave was established, subthreshold optical stimulation was delivered with varying parameters. The light intensity ranged from 0.004 to 1.01 µW mm^–2^, and illumination was applied as a uniform vertical rectangular strip starting from the left edge of the domain, with spatial coverage increasing from 0 to 100% in 10% increments from left to right. Different spatial intensity profiles were tested, including uniform, parabolic, linearly increasing and exponentially increasing distributions, to assess the impact of illumination pattern on termination efficiency. The spatial intensity profile of the illumination was defined as a function of the normalized distance from the centre of the domain (*x*, where 0  ≤  *x*  ≤  1). For non-uniform patterns, the intensity at each point is calculated using the following equations:

$$\begin{array}{rcl} linearly\;increasing & : & I_{lin(x)}=0.01+1.00x,\\ parabolic\;\left(quadratic\right) & : & I_{quad(x)}=1.01\times \left[1-4(x-0.5{)}^{2}\right] \end{array}$$

and

$$\begin{array}{r} exponentially\;increasing:I_{exp(x)}=0.01\times e^{4.605x}. \end{array}$$

These profiles were applied across the entire illuminated region. The maximum intensity (*I*_max_) was set to 1.01 µW mm^–2^ for all non-uniform patterns, ensuring that the peak light level remained consistent with the uniform illumination condition. All simulations are conducted under no-flux boundary conditions. Spiral wave termination is defined as the complete disappearance of electrical activity across the entire domain for a duration exceeding 300 ms.

### 2.6. Model validation and sensitivity analysis

To evaluate the robustness of our findings to model assumptions and numerical implementation, we conducted four complementary sensitivity analyses. Full results are provided in the supplementary material.

#### 2.6.1. Reversal potential of GtACR1

The GtACR1 reversal potential (*E*_GtACR1_) was varied across six physiologically plausible values (−80, −60, −50, −40, −30 and −20 mV) to span the range of chloride equilibrium potentials reported in cardiac myocytes. Spiral wave termination was simulated under global illumination (100% coverage) in two representative substrates: 50% diffuse and 50% focal fibrosis.

#### 2.6.2. Numerical convergence

A grid refinement study was performed to ensure solution independence. The baseline discretization used spatial step *dx* = 0.025 cm and temporal step d*t* = 10 µs. Three refined configurations were tested: (i) halved time step (d*t* = 5 µs), (ii) refined spatial grid (d*x* ≈ 0.0177 cm, corresponding to a $\sqrt{2}$ reduction in mesh area), and (iii) fully refined grid (d*x* ≈ 0.0177 cm, d*t* = 5 µs). Termination times were compared for reference cases (50% fibrosis and 100% illumination).

#### 2.6.3. Fibrosis-related parameters

Key assumptions regarding fibroblast–myocyte coupling and conduction slowing were perturbed by ± 50%: gap junction conductance G_gap_ was varied from 1.0 to 3.0 nS (baseline: 2.0 nS), and conductivity reductions in fibrotic regions were adjusted from (10%, 30%) to (5%, 15%) and (15%, 45%). Simulations covered illumination areas from 50 to 100% in both diffuse and focal 50% fibrosis models.

#### 2.6.4. Physiological realism

To assess the impact of model simplifications, we repeated key simulations at a clinically observed conduction velocity (approx. 70 cm s^–1^) by increasing the diffusion coefficient [30], and introduced 2 : 1 structural anisotropy via unequal spatial steps (d*x* = 0.05 cm and d*y* = 0.025 cm).

#### 2.6.5. Stochasticity of fibrosis spatial configuration

Multiple independent random realizations of fibrosis were generated for both diffuse and focal patterns to assess how random fluctuations in spatial distribution affect termination time and core dynamical phenomena.

Building on the original validation of spatial stochasticity of fibrosis, we further incorporated two additional dimensions of robustness testing. First, the domain size effect was evaluated by doubling the spatial dimensions to assess the impact of boundary-mediated annihilation mechanisms on core conclusions. Second, the frequency-dependent conduction effect of fibrosis was examined by reducing tissue conductivity in fibrotic regions to 70% of baseline under 10 Hz high-frequency pacing, in reference to the quantitative experimental findings of Giardini *et al.* [31], and compared against the 5 Hz low-frequency baseline to evaluate the stability of optogenetic termination rules at pathological heart rates. Both validations were performed with 10 independent random fibrosis realizations per condition, with the mean and standard deviation of termination time calculated for statistical analysis.

These analyses collectively test the dependence of optogenetic termination efficacy on biophysical, numerical and structural uncertainties.

## 3. Results

### 3.1. Action potential of human atrial myocytes and fibroblasts in the presence of GtACR1 regulation

**Figure 1.**
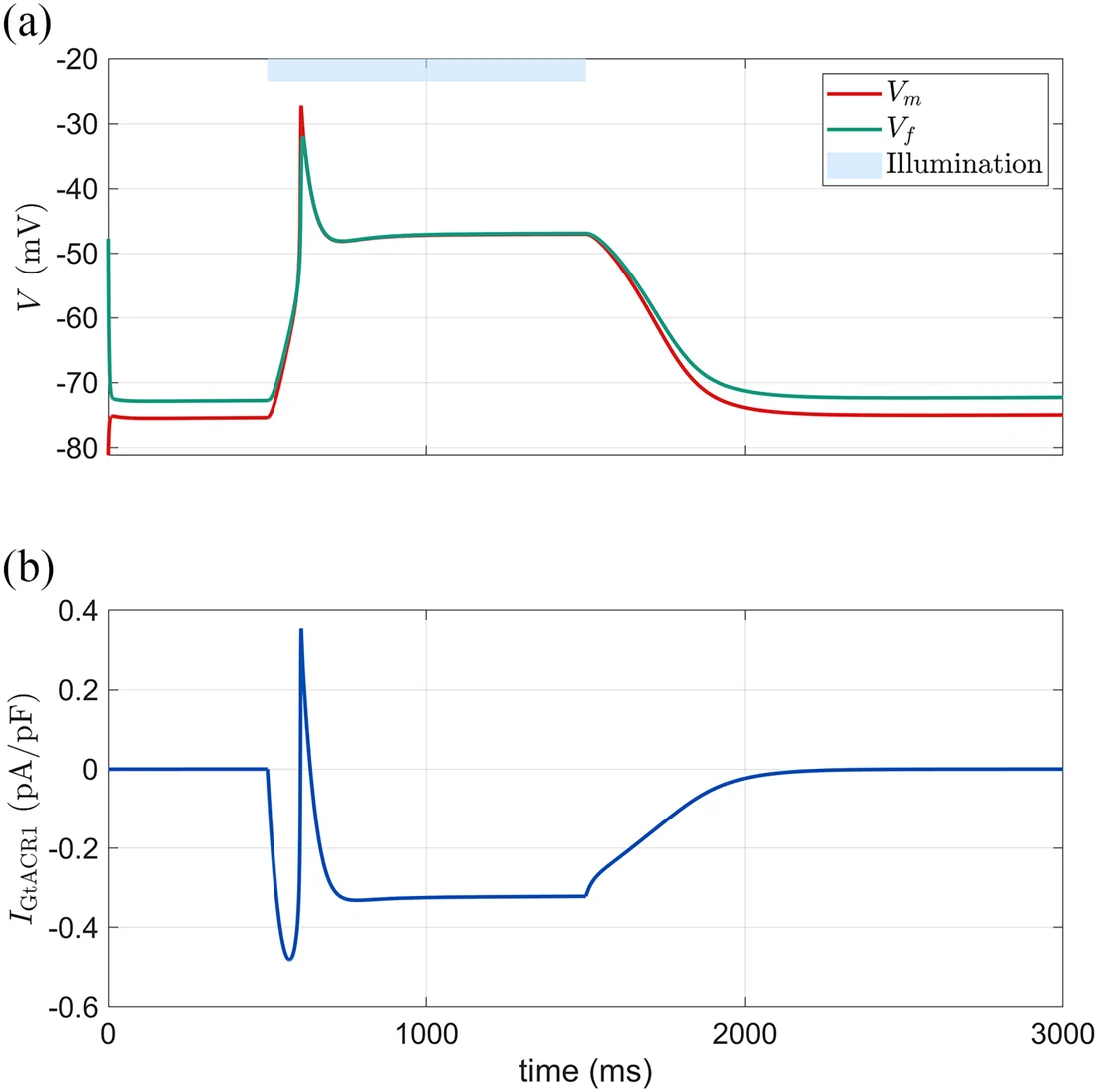
GtACR1-mediated electrophysiological responses in human atrial myocytes and fibroblasts. (a) Simulated action potentials of a GtACR1-expressing atrial myocyte (red) and a coupled fibroblast (green) during optical stimulation (light-blue bar: 500–1500 ms). (b) Time course of the GtACR1-mediated current, showing a triphasic response upon light activation.

Figure 1 illustrates the cellular-level responses of GtACR1-transduced human atrial myocytes and fibroblasts to optical stimulation. Figure 1a shows the simulated action potentials of an atrial myocyte (red trace) and a coupled fibroblast (green trace) under uniform illumination applied between 500 and 1500 ms (indicated by the light-blue bar). In the dark phase (before 500 ms), both cells maintain their respective resting potentials. Upon illumination, both undergo a rapid shift in membrane potential, transitioning to a sustained depolarized state centred around −40 mV. The fibroblast also exhibits a similar steady-state shift, reflecting direct modulation by GtACR1-mediated ion flux. Figure 1b presents the time course of the GtACR1-mediated transmembrane current. At light onset (500 ms), a brief initial inward current (approx. −0.5 pA) is observed, followed by a transient outward current (approx. +0.35 pA), and then a stable inward current that persists at approximately −0.3 pA for the duration of illumination. The presence of this light-evoked current correlates temporally with the altered membrane dynamics in both cell types, confirming functional expression of the opsin and its capacity to influence cellular electrophysiology.

### 3.2. Effect of terminating spiral waves in different illumination ranges in different fibrosis conditions

**Figure 2.**
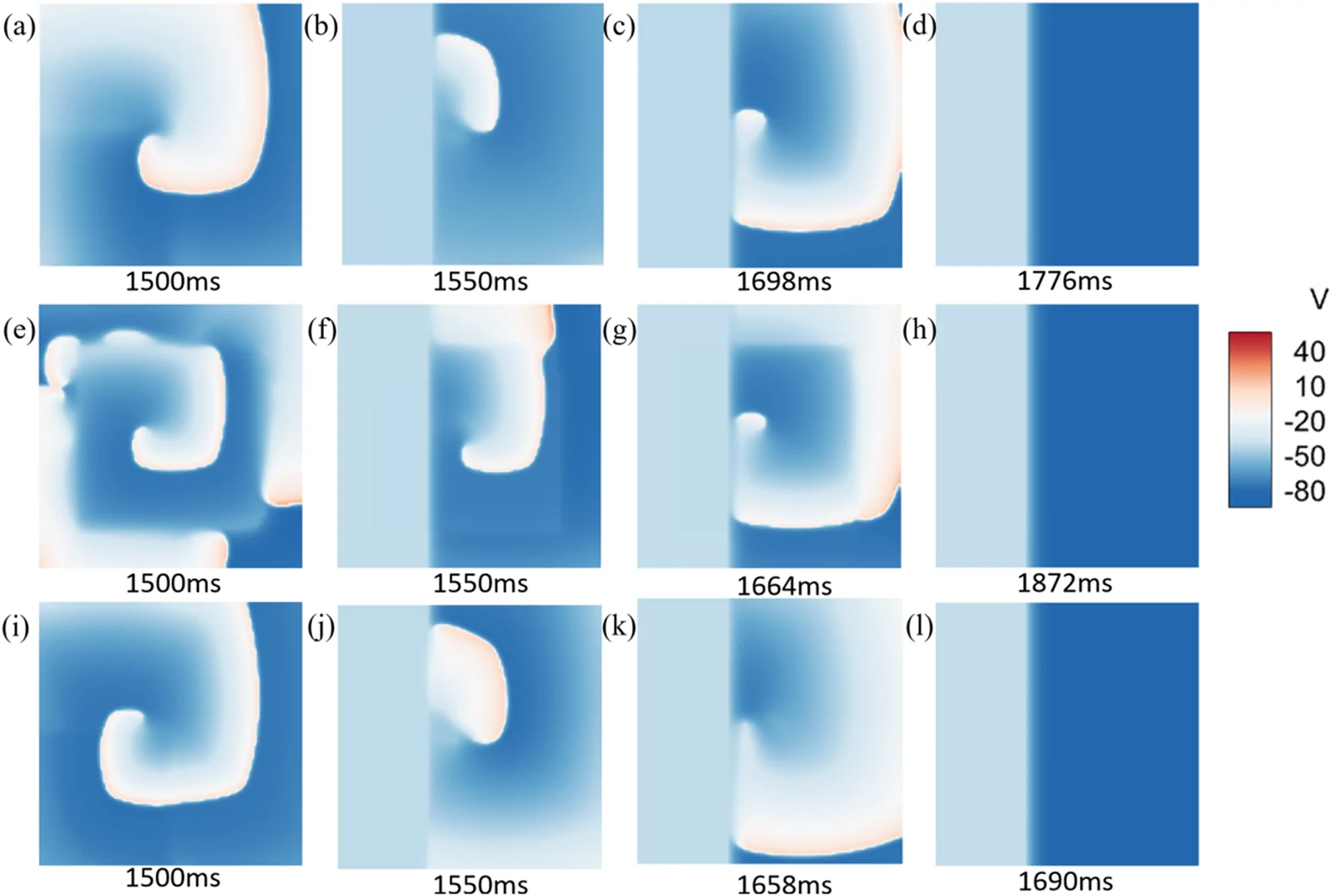
Spiral wave dissipation in atrial tissues under 35% light coverage (0.1 µW mm^–2^). (a–d) Spiral wave dissipation in pure atrial myocytes. (e–h) Spiral wave dissipation in focal atrial fibrosis (50% fibrosis). (i–l) Spiral wave dissipation in diffuse atrial fibrosis (50% fibrosis).

Figure 2 employed two-dimensional computer simulations to investigate the termination efficacy of the light-sensitive protein GtACR1 on spiral waves in atrial myocytes with varying fibrosis degrees (0, 25, 50, 75 and 100%) and distribution types (diffuse and focal) in different light ranges. At an illumination range of 35%, the results for pure atrial myocytes, 50% focal fibrosis (the fibrosis area is concentrated in the rectangular area at 50% centre of the two-dimensional plane) and 50% diffuse fibrosis are presented in figure 2.

**Figure 3.**
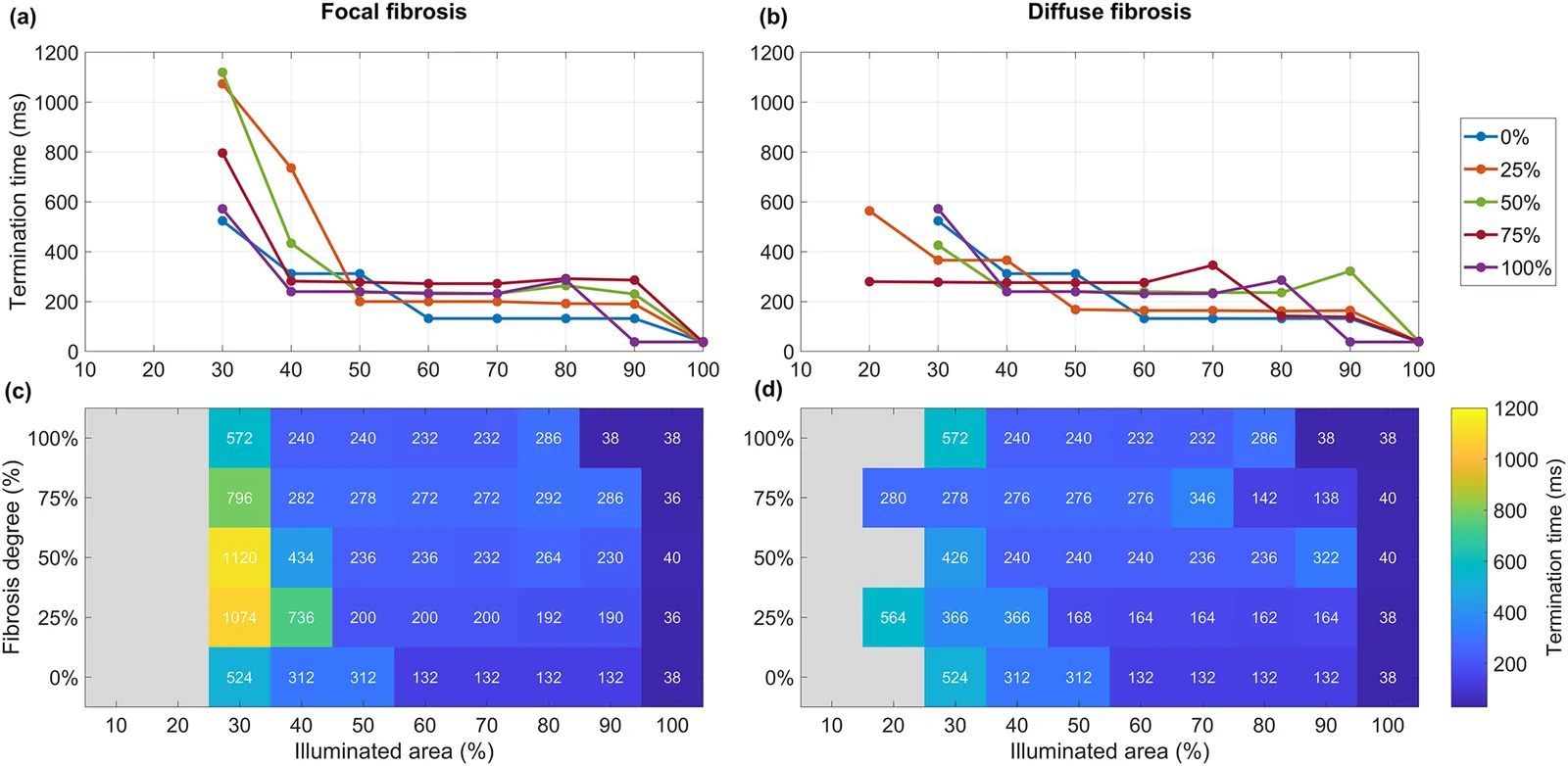
Relationship between spiral wave termination time and percentage of illuminated area in a two-dimensional atrial tissue sheet under constant light intensity (0.1 µW mm^–2^). (a) Focal fibrosis and (b) diffuse fibrosis. The x-axis represents the percentage of illuminated area, and the y-axis shows the time required for spiral wave termination. Different coloured curves correspond to various degrees of fibrosis (0%, 25%, 50%, 75% and 100%). (c) Heatmap representation of termination time across all combinations of fibrosis degree (y-axis) and illuminated area (x-axis) for focal fibrosis; grey regions indicate no successful termination within 3000 ms. (d) Same as (c) but for diffuse fibrosis.

Figure 2 illustrates the dissipation of spiral waves in a two-dimensional plane under 35% light coverage across three conditions: pure atrial myocytes, focal atrial fibrosis (50% fibrosis), and diffuse atrial fibrosis (50% fibrosis). Specifically, in pure atrial myocytes, spiral waves completely dissipate in 276 ms (figure 2a–d); in focal atrial fibrosis (50% fibrosis), spiral waves take 372 ms to fully dissipate (figure 2e–h); in diffuse atrial fibrosis (50% fibrosis), spiral waves dissipate in 190 ms (figure 2i–l). This result contradicts the expectation that spiral waves in pure atrial myocytes would dissipate the fastest. To further investigate this discrepancy, additional simulations were conducted for pure atrial myocytes and the two fibrosis types (diffuse and focal) at varying degrees of fibrosis (0%, 25%, 50%, 75% and 100%) and different light coverage areas (0–100% of the plane, increasing from left to right). The termination times of spiral waves under these conditions were calculated. The results were illustrated in figure 3.

In focal fibrosis conditions, the overall trend shows a decrease in spiral wave termination time as the illuminated area increases. For 25% fibrosis (orange line), measurable termination begins when illumination coverage exceeds 45% with a termination time of 200 ms. Between 45 and 80% illumination, this time remains stable between 190 and 200 ms before gradually decreasing further with increased illumination, ultimately reaching 36 ms under full coverage. In 75% fibrosis (red line), effective termination starts at 40% illumination (282 ms) with termination times between 278 and 292 ms across the 40–80% illumination range, then decreases to 36 ms at full illumination. For 100% fibrosis (purple line), there is an anomalous increase in termination time to 286 ms at 80% illumination, followed by a rapid decline to 38 ms at 90% illumination, stabilizing thereafter.

In diffuse fibrosis conditions, the overall trend also shows a reduction in termination time with increasing illuminated area. For 50% fibrosis (green line), termination time remains stable at approximately 240 ms across the 40–80% illumination range; at 90% illumination, it anomalously rises to 322 ms before rapidly declining to stabilize at 40 ms under full coverage. For 75% fibrosis (red line), the termination time stays around 278 ms from 20 to 60% illumination, rising to 346 ms at 70% illumination before quickly dropping back to 40 ms at full illumination. In 100% fibrosis (purple line), the termination time ranges around 232–240 ms between 40 and 70% illumination, increasing to 286 ms at 80% illumination, then sharply decreasing to stabilize at 38 ms under full illumination.

Comparing the two types of fibrosis reveals that spiral waves in diffuse fibrotic substrates can be terminated with smaller illuminated areas compared with those in focal fibrosis. For example, the red curve (75% fibrosis) shows effective intervention starting at 20% illumination in the diffuse model, whereas in the focal model, effective termination requires at least 40% coverage.

The accompanying heatmaps in figure 3c,d provide a comprehensive overview of termination efficiency across the full parameter space of fibrosis degree and illumination coverage. They clearly visualize the higher sensitivity of diffuse fibrosis to optical intervention, as successful termination occurs at smaller illuminated areas across all fibrosis levels, and further highlight the non-monotonic ‘optimal range’ phenomenon, which is particularly evident at 70–80% illumination in moderate-to-severe diffuse fibrosis.

**Figure 4.**
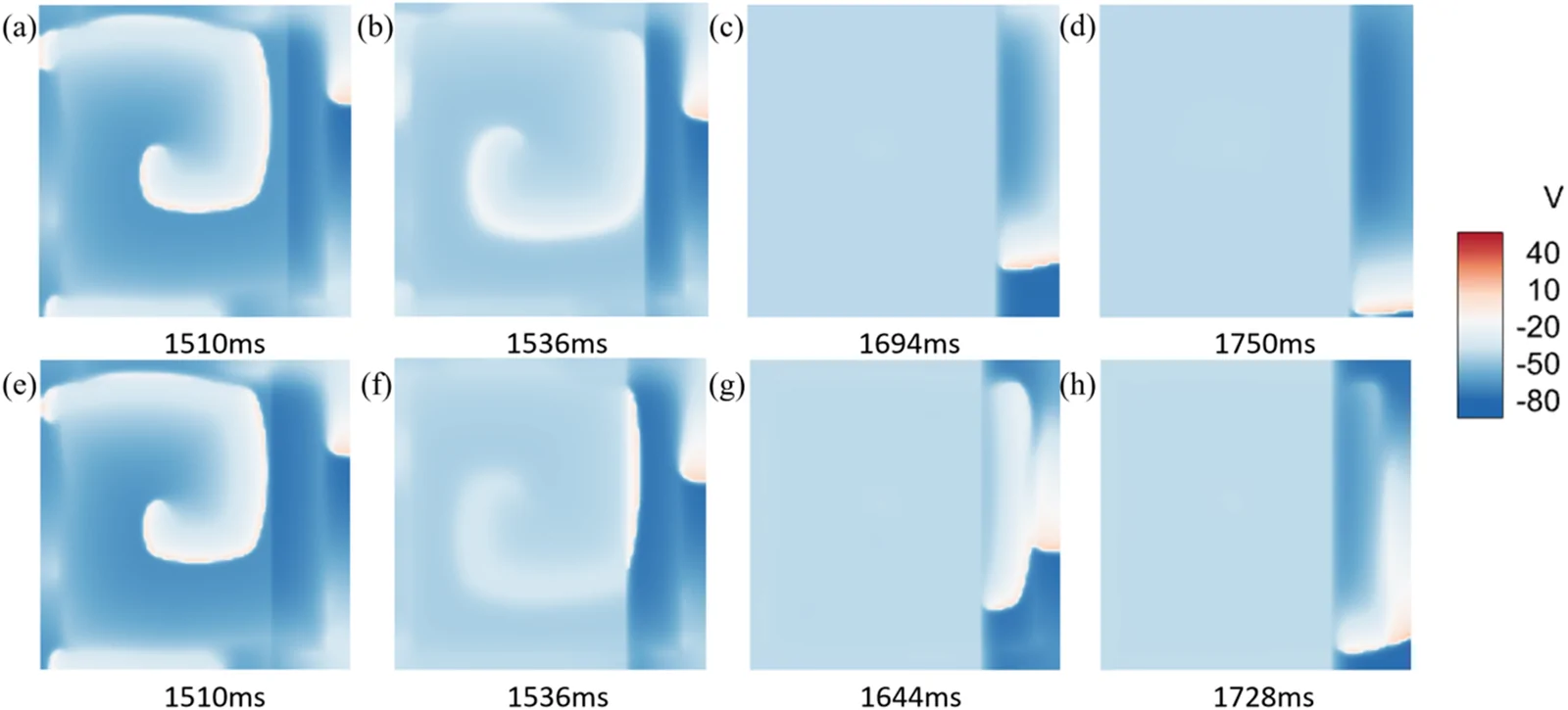
Collision-induced acceleration of spiral wave dissipation in focal fibrotic atrial tissue under suboptimal illumination (0.1 µW mm^–2^). (a–d) 80% illumination: complete suppression of the main spiral wave, leaving a slowly dissipating fragmented wave. (e–h) 75% illumination: residual wavefronts collide with fragmented waves, accelerating annihilation.

Across all tested conditions, the non-monotonic response is consistently observed in both diffuse and focal fibrosis, but the underlying dynamical processes differ markedly. In diffuse fibrosis, the spiral wave remains as a single intact rotor throughout the entire termination process, with no spontaneous wavebreak or multi-wavefront collision detected at any illumination level. In focal fibrosis, by contrast, partial coverage of the central fibrotic core may split the main rotor into a small number of wave fragments, with rare collision events observed only at intermediate coverage ratios. To illustrate the fragment interaction pathway specific to focal fibrosis, we selected a representative case for detailed analysis, as shown in figure 4.

To characterize the fragment interaction pathway specific to focal fibrosis under intermediate illumination, we selected a representative case for simulation analysis. Figure 4 presents dynamic snapshots of spiral wave evolution in a 75% fibrotic atrial tissue with focal fibrosis under 80% (a–d) and 75% (e–h) illumination coverage. It is observed that under 75% illumination, the spiral wave is not fully encompassed by the illuminated region; residual wavefronts persist outside the right boundary of the light-exposed area. These uncovered wavefronts continue to propagate and collide with fragmented wavelets remaining in the upper-right region of the tissue, subsequently accelerating propagation toward the lower-right corner, coalescing into a single wavefront and ultimately dissipating. By contrast, under 80% illumination, the main body of the spiral wave is completely covered and rapidly suppressed, leaving only a small fragmented wave in the upper-right region to propagate into the non-illuminated zone before gradually dissipating. Compared with the 75% illumination condition, the 80% illumination scenario lacks wavefront fusion, and the dissipation time of the isolated fragmented wave is prolonged by approximately 26 ms.

To further quantify the dynamical features of spiral wave termination under different fibrosis patterns, we calculated core metrics including wavebreak events, phase singularities, wavefront collisions, drift velocity and boundary distance across the termination process, as summarized in table 1. All metrics are computed from the onset of illumination until complete electrical quiescence across the entire domain. The results show that no new spontaneous wavebreak events are induced by illumination under all tested conditions, with a wavebreak rate of zero throughout. The peak wave fragment count only reflects pre-existing steady-state fragments caused by fibrotic conduction heterogeneity and residual segments produced by illumination-induced cleavage, with no secondary wavebreak leading to turbulent transition. The peak number of phase singularities remains one in all cases, indicating that the termination process is consistently dominated by a single rotating rotor, and straight wave fragments do not develop into new stable re-entrant circuits. For diffuse fibrotic substrates, both the mean drift velocity of the dominant wavefront and the mean distance to the nearest boundary decrease monotonically with increasing illumination ratio, reflecting progressively strengthened geometric confinement caused by narrowing of the excitable region. For focal fibrotic substrates, the peak fragment count and wavefront collision frequency are higher at intermediate illumination ratios, corresponding to the fragment fusion accelerated annihilation phenomenon observed in figure 4. When the illumination ratio exceeds 80%, both fragment number and collision frequency decline simultaneously, and the termination process shifts to a boundary annihilation mode in which a single residual wave propagates along the narrow strip.

**Table 1.** Summary of quantitative dynamical metrics of spiral wave termination under different fibrosis patterns and illumination ratios.

**fibrosis pattern**	**illumination ratio (%)**	**termination time (ms)**	**peak wave fragment count**	**wavebreak rate (events per 100 ms)**	**peak phase singularity count**	**total wavefront collision events**	**mean drift velocity of dominant wavefront (cm s^–1^)**	**mean distance to nearest excitable boundary (cm)**
50% diffuse fibrosis	50	230	1	0	1	2	15.8	0.21
	70	226	1	0	1	2	10.7	0.16
	90	330	1	0	1	2	6.8	0.10
	100	38	1	0	1	0	—	—
75% focal fibrosis	40	282	5	0	1	2	9.8	0.26
	60	272	4	0	1	2	10	0.19
	75	274	4	0	1	2	10.1	0.14
	80	292	4	0	1	1	8.7	0.11
	100	36	5	0	1	0	—	—

*Note:* All metrics are calculated from the onset of illumination until complete electrical termination. Peak wave fragment count represents the maximum number of independently connected excitation units during the termination process, including pre-existing steady-state fragments formed by fibrotic conduction heterogeneity. No new spontaneous wavebreak events are generated after illumination onset. Only rotating spiral cores are counted as phase singularities, and linear travelling wave fragments are excluded. Drift velocity and boundary distance are measured based on the innermost leading edge of the dominant excitation wave, covering both the spiral rotor phase and the travelling wave phase after the phase singularity is suppressed. Boundary distance is defined as the minimum distance from the wavefront to the nearest excitable boundary, averaged over all valid frames. The symbol ‘—’ indicates no valid drift trajectory owing to rapid global suppression under 100% illumination.

### 3.3. Influence of different light intensity distribution patterns on the termination effect of spiral waves

**Figure 5.**
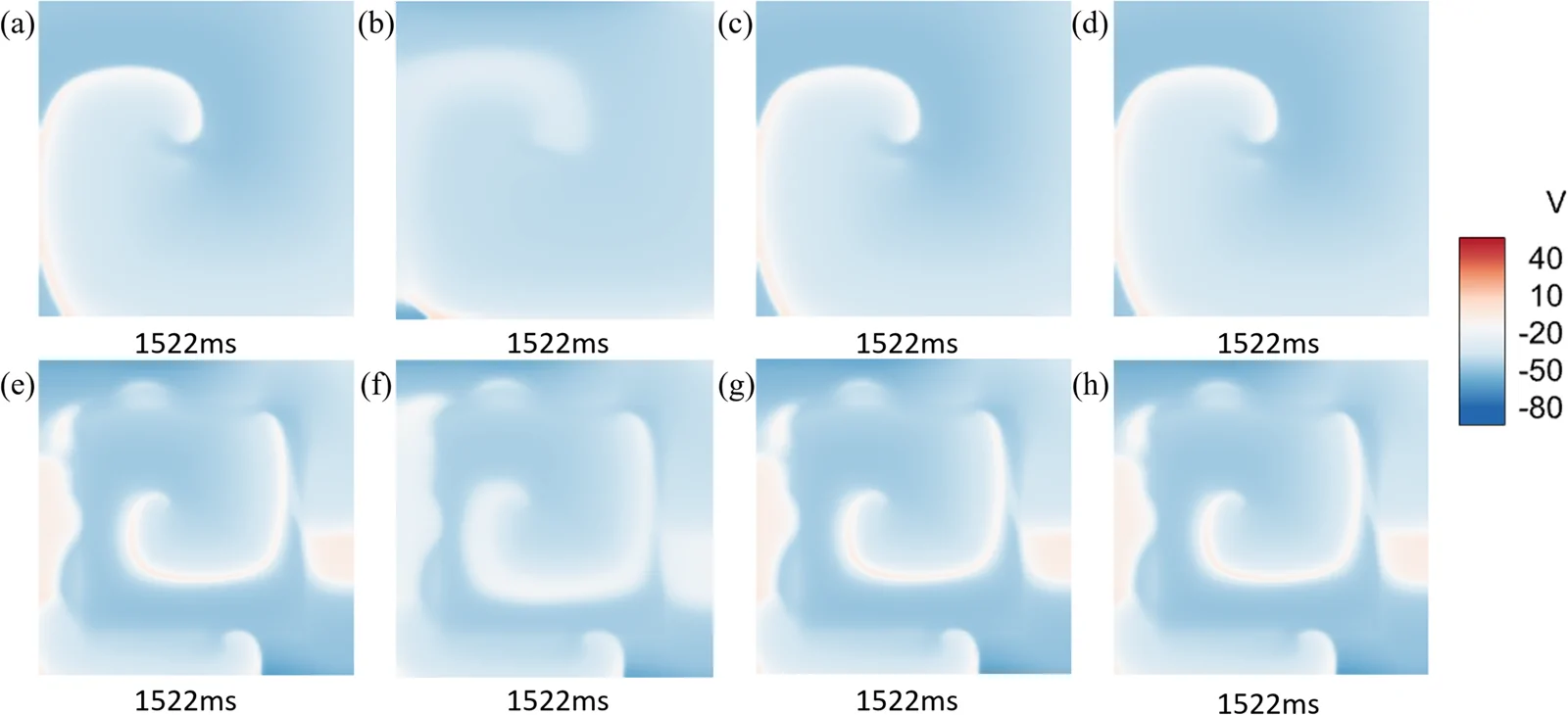
Effect of light intensity distribution on spiral wave dynamics in atrial tissue with diffuse and focal fibrosis under full-field illumination. (a and e) Uniform illumination (1 µW mm^–2^) on diffuse fibrosis (a) and focal fibrosis (e). (b and f) Parabolic illumination on diffuse fibrosis (b) and focal fibrosis (f). (c and g) Linearly increasing illumination from left to right on diffuse fibrosis (c) and focal fibrosis (g). (d and h) Exponentially increasing illumination from left to right on diffuse fibrosis (d) and focal fibrosis (h).

After investigating the effect of illumination coverage area on spiral wave termination, we further explored the influence of different light intensity distribution patterns on spiral wave dynamics in a two-dimensional domain. Figure 5 presents the evolution of spiral waves under four distinct light intensity profiles with full-field illumination coverage. The top row shows results for atrial tissue with diffuse fibrosis (a–d), and the bottom row for focal fibrosis (e–h), with both substrates having a fibrosis level of 50%. The four columns represent the following illumination patterns from left to right: (a and e) uniform illumination at 1.01 µW mm^–2^ across the entire domain; (b and f) parabolic illumination with peak intensity of 1.01 µW mm^–2^ at the centre, tapering to 0 µW mm^–2^ at both lateral edges; (c and g) linearly increasing illumination from 0 µW mm^–2^ on the left edge to 1.01 µW mm^–2^ on the right edge; and (d and h) exponentially increasing illumination from 0 µW mm^–2^ on the left to 1.01 µW mm^–2^ on the right. Visually, no significant differences in spiral wave dynamics are observed across the four illumination patterns. The time required for spiral wave dissipation under each condition was recorded (table 2). The data show that, for both fibrotic substrates at the same fibrosis level, the termination times are nearly identical across all four illumination modes. This indicates that, under full-field illumination coverage, variations in the spatial distribution of light intensity do not significantly affect spiral wave termination.

**Table 2.** Termination duration of spiral waves under different illumination patterns, fibrosis levels and tissue types (unit: ms).

**illumination pattern**	uniform illumination	parabolic illumination	linearly increasing illumination	exponentially increasing illumination
diffuse (0%)	38	38	38	38
focal (0%)	38	38	38	38
diffuse (25%)	38	36	36	36
focal (25%)	36	38	38	38
diffuse (50%)	40	38	38	38
focal (50%)	40	40	38	38
diffuse (75%)	38	38	38	38
focal (75%)	36	38	40	38
diffuse (100%)	38	38	38	38
focal (100%)	38	38	38	38

### 3.4. Effect of low-intensity periodic light on spiral wave trajectory in fibrotic atrial tissue

**Figure 6.**
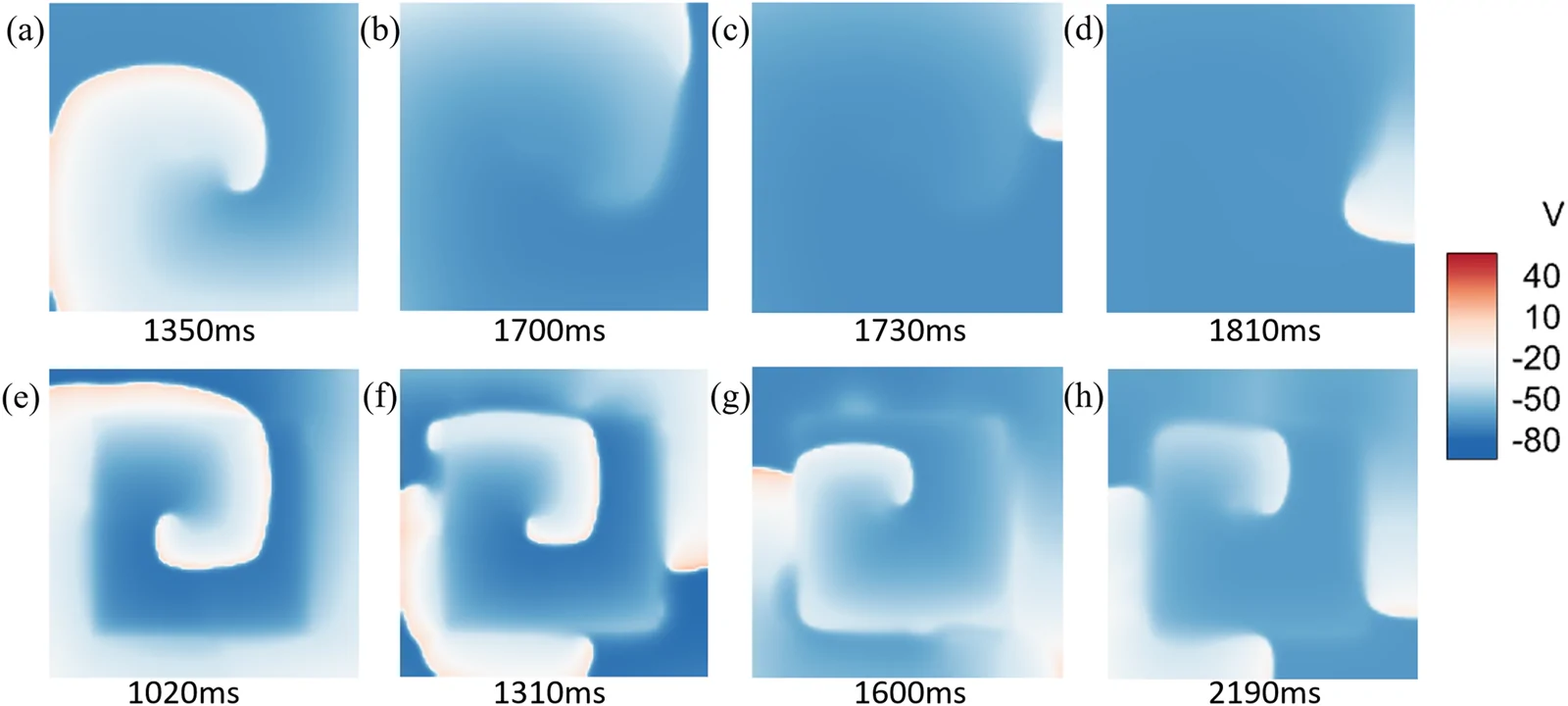
Spiral wave dynamics under periodic low-intensity illumination (0.004 µW mm^–2^) in atrial tissue with 50% diffuse and focal fibrosis. (a–d) Diffuse fibrosis: the spiral wave meanders, drifting first toward the upper-right and then downward. (e–h) Focal fibrosis: the spiral wave remains anchored near the centre with no significant drift.

**Figure 7.**
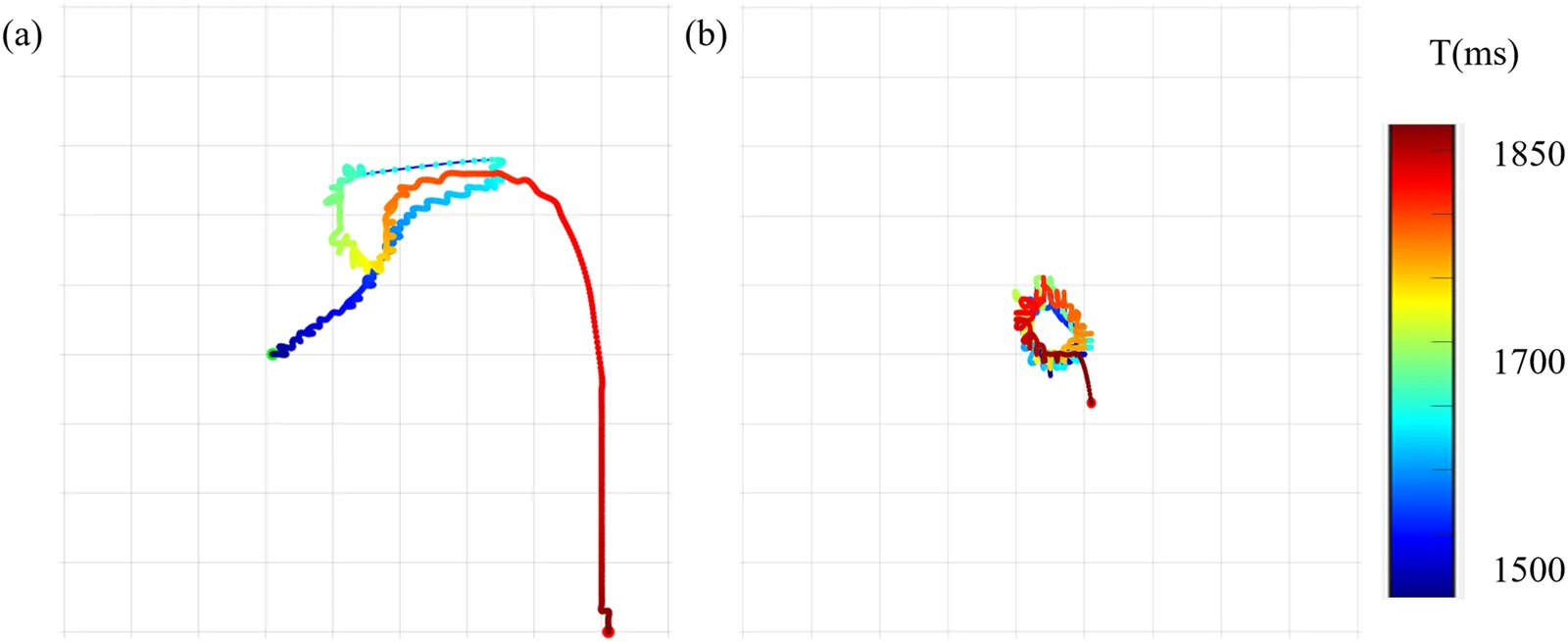
Trajectories of spiral wave tips under periodic low-intensity illumination (0.004 µW mm^–2^) in 50% fibrotic atrial tissue. (a) In diffuse fibrotic tissue, the wave tip follows a meandering path, eventually reaching the boundary and annihilating. (b) In focal fibrotic tissue, the wave tip remains confined to the central region without displacement.

Although we have previously established that a light intensity of 0.1 µW mm^–2^ is sufficient to terminate spiral waves in GtACR1-transduced atrial tissue with 50% fibrosis (figure 3), we further investigated the effects of periodic low-intensity global illumination to minimize potential photodamage. In this protocol, both the illumination duration and the dark interval were set to half the rotation period of the spiral wave (approx. 190 ms), resulting in repeated cycles of 95 ms of global uniform illumination followed by 95 ms of darkness. Figure 6 illustrates the spiral wave dynamics under this illumination scheme in atrial tissue monolayers with diffuse fibrosis (figure 6a–d) and focal fibrosis (figure 6e–h), both at 50% fibrosis density. In diffuse fibrotic tissue, the spiral wave exhibits meandering motion—the wave tip initially drifts toward the upper-right corner, then shifts downward. By contrast, in focal fibrotic tissue, the spiral wave remains anchored near the centre without significant drift. The wave tip trajectories under this periodic low-intensity illumination are shown in figure 7. In diffuse fibrosis (figure 7a), the wave tip moves from the centre toward the upper-right region, then loops back to the left, approaches the centre again, drifts upward once more and finally propagates straight downward to the tissue boundary, where it annihilates. In focal fibrotic tissue (figure 7b), the wave tip remains confined to the central region with no notable displacement.

### 3.5. Robustness of key findings

To ensure the robustness of our conclusions, we performed a comprehensive set of sensitivity and validation analyses (see §2). We first assessed the variability of termination time across independent stochastic realizations of fibrosis to rule out biases introduced by a single random configuration. Replicate simulations for both diffuse and focal fibrosis patterns across four coverage levels are presented in figures S1 and S2 and table S7 in the supplementary material. Figure S1 in the supplementary material provides an overview of overall trends with mean and standard deviation values for both fibrosis patterns, while figure S2 in the supplementary material displays the full distribution of individual replicates for each condition in faceted scatter plots. Across all tested conditions, the within-group standard deviation of termination time is substantially smaller than the between-group differences driven by fibrosis percentage or illumination area. For a representative condition of 50% diffuse fibrosis with 50% illumination coverage, the mean termination time across 10 random realizations is 233.8 ms, with a standard deviation of only 9.82 ms, corresponding to a coefficient of variation below 5%. For the more spatially constrained focal fibrosis, standard deviations are generally below 2 ms. Importantly, the core dynamical observations are highly reproducible across realizations. The non-monotonic peak in termination time at 70% illumination in 75% diffuse fibrosis, for instance, is consistently observed across all 10 independent configurations, with a within-group standard deviation of only 6.85 ms and a coefficient of variation of approximately 1.9%. These results confirm that our core findings, namely the lower termination threshold of diffuse fibrosis and the non-monotonic relationship between termination time and illumination coverage are robust to the specific random realization of the fibrotic substrate.

We further verified the influence of computational domain size and frequency-dependent conduction on our core conclusions. Control experiments with doubled domain size revealed highly consistent trends in termination time versus illumination area, with the optimal illumination window remaining stably within the 40–80% coverage range and showing no obvious shift (supplementary material, figure S3 and table S8). Only absolute termination times increased globally by 170–200 ms owing to extended wave propagation paths, and the amplitude of the non-monotonic rise at 90% coverage was slightly attenuated, indicating that boundary effects modulate only the quantitative magnitude without altering the dynamical nature of the phenomenon. Validation of frequency-dependent conduction demonstrated fully consistent patterns under low-frequency conditions (supplementary material, figure S4 and table S9). Under high-frequency pacing, overall termination times were universally prolonged, and a clear dynamical bifurcation emerged at 70% illumination coverage, with a subset of random realizations showing elevated termination times, owing to wavebreak and extra excitation waves, resulting in a markedly increased standard deviation at this condition. The 40–60% illumination range maintained stable low termination time and low dispersion even at high frequency, representing the most reliable termination interval across pacing rates.

We evaluated the sensitivity of spiral wave termination dynamics to the *E*_GtACR1_and numerical discretization under global uniform illumination. As summarized in tables S1 (50% focal fibrosis) and S2 (50% diffuse fibrosis) in the supplementary material, spiral waves were successfully terminated across all tested *E*_GtACR1_ values (−80 to −20 mV). However, termination time exhibited a pronounced dependence on *E*_GtACR1_—it generally decreased as *E*_GtACR1_ became more depolarized, with a notable maximum observed at *E*_GtACR1_= −60 mV in both fibrotic substrates. Importantly, this qualitative trend was preserved across all numerical resolutions. The absolute variation in termination time owing to refinement of spatial and temporal discretization was minimal and did not exceed 30 ms in both fibrosis.

Tables S3 and S4 in the supplementary material demonstrate that while spiral wave termination dynamics are insensitive to global variations in gap junction conductance, they are highly sensitive to the degree of fibrosis-induced intercellular conductance reduction. Specifically, stronger conduction slowing in fibrotic regions (modelled by 15%/45% reductions) markedly prolongs termination times, particularly in focal fibrosis. Nevertheless, the qualitative conclusions of our study—namely, the superior controllability of diffuse fibrosis and the non-monotonic dependence on illumination coverage—remain robust across all tested parameter combinations.

The non-monotonic dependence of spiral wave termination time on illumination coverage, and the overall responsiveness of diffuse fibrotic substrates to optogenetic intervention were preserved under extended modelling conditions. At a conduction velocity of approximately 70 cm s^–1^ in 50% diffuse fibrosis (supplementary material, table S5), termination times ranged from 102 to 178 ms across 50–80% illumination, with a local maximum at 80% coverage (178 ms), before decreasing to 46 ms at 90% and 38 ms at 100% illumination. This pattern mirrors the non-monotonic trend observed at the baseline conduction velocity (43.9 cm s^–1^). Under anisotropic tissue conditions (d*x* = 2d*y*), simulations across 50%, 75% and 100% diffuse fibrosis (supplementary material, table S6) showed consistently longer termination times at intermediate illumination levels compared with isotropic cases. For example, in 75% fibrosis, termination times under 50–70% illumination ranged from 480 to 672 ms, with a peak of 672 ms at 70% coverage. In all fibrosis degrees, however, termination times decreased sharply when illumination exceeded 80%, reaching 38–40 ms at full coverage—comparable to isotropic results. Thus, both increased conduction velocity and structural anisotropy altered the absolute magnitude of termination times, but did not abolish the characteristic non-monotonic response to illumination area or the efficacy of complete tissue illumination.

## 4. Discussion and conclusion

This study establishes that the spatial patterning of atrial fibrosis, either diffuse or focal, serves as a primary determinant of optogenetic spiral wave control in GtACR1-expressing human atrial tissue. Diffuse fibrosis permits efficient termination through either modest illumination coverage or low-intensity periodic stimulation, both of which can induce self-terminating drift toward tissue boundaries. By contrast, focal fibrosis enforces robust rotor anchoring that resists drift and necessitates extensive high-intensity illumination for complete annihilation. Moreover, we uncover a counter-intuitive yet consistent principle that termination time exhibits a non-monotonic dependence on illuminated area. An optimal range, typically between 40 and 70% coverage, maximizes annihilation efficiency through geometric reshaping of the excitable domain and fragment interactions where applicable, while excessive coverage above 80% paradoxically prolongs persistence by eliminating wavefront interactions and imposing narrow-strip geometric confinement. Notably, under full-field illumination, termination becomes insensitive to spatial light intensity profiles, underscoring that global excitability suppression rather than local intensity gradients drives final wave collapse. Critically, these findings are not contingent on idealized model assumptions and persist across a comprehensive set of sensitivity analyses spanning opsin biophysics, numerical resolution, fibrosis-induced conduction slowing, clinically relevant conduction velocity and structural anisotropy, all of which are documented in tables S1–S9 in the supplementary material. With this multi-scale robustness confirmed, we interpret these phenomena through the framework of nonlinear wave dynamics to elucidate the mechanistic interplay between fibrotic substrate topology and spatially targeted optical perturbation.

### 4.1. The critical role of fibrosis spatial pattern in optogenetic control efficiency

In the field of optogenetic virtual heart modelling, various strategies have been developed to terminate spiral waves through light stimulation [32–34]. Studies have also demonstrated that in optogenetically modified cardiac tissue, regional illumination can induce directional linear drift of spiral waves [35,36]. For instance, Hussaini *et al.* [37] showed in a two-dimensional model of adult mouse ventricles that localized illumination effectively controls the direction of spiral wave drift, which consistently moves towards the region of increasing light intensity. However, these studies are predominantly based on healthy or homogeneous myocardial tissue models, with limited consideration of the structural heterogeneity associated with atrial fibrosis—a common clinical pathological substrate. Our study reveals the decisive influence of fibrosis spatial distribution (diffuse versus focal) on the efficiency of optical control. Under 40% illumination coverage, spiral wave termination time is shortest in diffusely fibrotic tissue (240 ms), longest in focally fibrotic tissue (736 ms) and intermediate in non-fibrotic myocardium (312 ms). The underlying mechanism lies in the fact that diffuse fibrosis, characterized by randomly distributed fibroblasts, enhances global conduction heterogeneity, rendering the spiral wave electrophysiologically unstable and thus more susceptible to local optical perturbation. By contrast, focal fibrosis forms a high-density structural barrier in the central region, anchoring the spiral wave core and significantly enhancing its rotational stability, thereby increasing resistance to optogenetic intervention.

### 4.2. Differential thresholds for effective illumination in diffuse and focal fibrotic substrates

Although optogenetic induction of spiral wave drift has been demonstrated, systematic evaluation of the minimal effective illumination area across different fibrosis types remains lacking [37,38]. In our simulations, spiral waves in diffusely fibrotic tissue exhibit higher sensitivity to low-coverage illumination, enabling effective termination with smaller illuminated areas, whereas focal fibrosis requires substantially more extensive coverage. For example, at 75% fibrosis, the diffuse model responds to inter vention at just 20% illumination, while the focal model requires at least 40% coverage. This difference arises from distinct electrophysiological response mechanisms—in diffuse fibrosis, localized illumination generates steep repolarization gradients at the boundary between illuminated and non-illuminated regions, inducing conduction block or depolarization that disrupts the spiral wave structure. In focal fibrosis, however, the spiral wave core is surrounded by a dense cluster of fibrotic tissue, forming an electrophysiological ‘safe zone’ that limits light penetration. Consequently, external illumination must be expanded to block the rotational pathway, necessitating broader spatial coverage for effective termination.

### 4.3. Non-monotonic dependence of termination time on illuminated area

Our study reveals that termination time does not decrease monotonically with increasing illumination area; instead, a clear non-monotonic optimal window emerges in both diffuse and focal fibrosis. This shared phenomenology arises from distinct dynamical mechanisms, both rooted in geometric reshaping of the functional excitable domain by left-to-right vertical strip illumination.

#### 4.3.1 Mechanism in diffuse fibrosis

In diffuse fibrosis, the non-monotonic effect is driven entirely by geometric constraints on the excitable domain, with no involvement of wavebreak or wavefront collision. Quantitative results from table 1 show that the wavebreak rate is zero at all illumination ratios, and only one phase singularity exists throughout the process. There is no generation of new independent rotors or interaction between fragments, which rules out any contribution of wavebreak dynamics to termination efficiency. Inhibitory illumination via GtACR1 renders the targeted vertical strip electrically inexcitable, and the boundary between illuminated and non-illuminated tissue acts as a functional insulating barrier that wavefronts cannot cross. As illumination coverage expands from left to right, the remaining excitable tissue on the right forms a vertical strip of decreasing width, and the efficiency of spiral wave annihilation at the boundary varies non-monotonically with strip width.

At low coverage below 40%, the excitable strip is wide, and the spiral rotor has sufficient space for free meandering. As indicated in table 1, the mean distance from the dominant wavefront to the nearest boundary is large, leading to a long waiting time for boundary encounter and relatively low termination efficiency. As coverage increases into the optimal range of 40 to 80%, the excitable strip narrows moderately and compresses the lateral drift space of the rotor. The mean distance to the nearest boundary decreases markedly, and the spiral core reaches the right physical boundary after a short drift path, resulting in fast and robust annihilation. This represents the most efficient termination regime.

When coverage further increases to 80–90%, the excitable strip becomes extremely narrow. The lateral space is no longer sufficient to support free transverse drift of the full rotor, and the wave is forced to propagate primarily along the longitudinal axis of the strip. In this regime, the mean drift velocity of the dominant wavefront drops to 6.8 cm s^–1^, and the average distance from the wave core to the upper or lower boundary is substantially longer than the transverse distance in the optimal window. Annihilation, therefore, takes longer, producing the paradoxical rise in termination time. At 100% global illumination, all tissue is synchronously clamped near the GtACR1 reversal potential, and re-entrant activity is eliminated immediately. This geometric mechanism is fully consistent with classical excitable medium theory, which predicts that rotor lifetime varies non-monotonically with domain size and aspect ratio. It also explains why the non-monotonic phenomenon persists across all degrees of diffuse fibrosis without requiring wave fragmentation or collision events.

#### 4.3.2 Mechanism in focal fibrosis

In focal fibrosis, the non-monotonic response arises from a combination of rotor anchoring–unbinding transitions and geometric confinement of residual fragments, with wavefront collision playing only a secondary and non-essential role. Quantitative data from table 1 demonstrate that the peak number of phase singularities remains at one throughout the process, confirming the absence of multi-rotor turbulence and that all fragments are straight-wave segments without rotational capacity.

At low coverage below 40%, the illumination strip does not reach the central fibrotic anchor. The spiral core remains stably anchored, and termination either fails or requires extremely long durations. Once coverage is sufficient to partially disrupt the anchor, corresponding to the effective interval of 40–80%, the main rotor unbinds and breaks into four to five wave fragments. According to table 1, the total number of wavefront collision events in this interval is two, including one fragment fusion event and one boundary annihilation event. Fragment fusion occurs only in a minority of random configurations and accelerates final annihilation by approximately 20 ms. In most configurations, only a single fragment remains and terminates via boundary encounter without any collision. Overall, disruption of the anchored rotor is the primary driver of reduced termination time in this regime, and collisions make a minor supplementary contribution to acceleration.

At high but sub-global coverage of 80–90%, the main rotor is fully suppressed, and only one small isolated fragment remains in the narrow right excitable strip. Deprived of wave–wave interactions, the fragment propagates along the long vertical axis of the strip and requires more time to reach the upper or lower corner for annihilation, leading to elevated termination time. This prolongation is driven by the same narrow-strip geometric constraint observed in diffuse fibrosis, rather than by loss of collisions. Notably, the mean drift velocity of the dominant wavefront drops to 8.7 cm s^–1^, and the mean distance to the nearest boundary decreases further in this regime, which further validates the dynamical feature of edge-hugging propagation and extended longitudinal path under narrow-strip confinement. Despite differing mechanistic origins between the two fibrosis patterns, the non-monotonic optimal window emerges consistently across all tested parameter conditions, indicating that it is a robust emergent property of structurally heterogeneous excitable media under localized inhibitory perturbation.

### 4.4. Uniform illumination suffices for global termination

Furthermore, studies suggest that global high-intensity illumination may be required for optogenetic pacing to fill excitable gaps, highlighting an essential distinction in illumination requirements between different control objectives (e.g. pacing versus defibrillation) [39]. In this study, focusing on defibrillation, we find that when full-field coverage is achieved, different spatial intensity profiles (e.g. uniform, parabolic, linear or exponential gradients) have no significant impact on spiral-wave termination efficiency. As long as the light intensity reaches the effective inhibition threshold (0.1 µW mm^–^²), the entire myocardial tissue synchronously enters a hyperpolarized state, blocking all excitable pathways. This indicates that termination efficiency primarily depends on whether the minimum effective intensity covers the entire domain, rather than on local intensity gradients. Therefore, under subthreshold conditions, spatial variations in light intensity contribute negligibly to overall suppression, supporting the use of simple, uniform illumination in clinical applications.

### 4.5. Structural constraints on spiral wave drift in anchored rotors

Hussaini *et al.* [37] observed that localized illumination can guide spiral wave drift, but their model did not account for structural anchoring effects induced by fibrosis. Our simulation results reveal distinct responses to periodic low-intensity illumination (0.004 µW mm^–^², 95 ms on/95 ms off) in different fibrotic substrates: in diffuse fibrosis, the spiral wave exhibits significant drift and eventually annihilates at tissue boundaries; and in focal fibrosis, the wave remains anchored to the fibrotic cluster and shows no substantial displacement. The mechanism lies in the differential response of anchored rotors to resonance: low-intensity periodic illumination can resonate with the intrinsic rotation frequency of the spiral wave, gradually pushing it outward in diffuse substrates, where the tissue matrix is relatively uniform, and drift resistance is low, allowing the wave core to migrate toward the boundary. In focal substrates, however, structural anchoring creates a strong ‘tethering effect’ that firmly fixes the wave core, preventing effective drift. This demonstrates that structural anchoring poses a major barrier to low-intensity, non-invasive interventions. For such patients, sustained high-intensity illumination is required to directly eliminate the anchor. Our findings emphasize that clinical translation must adopt a ‘tailored’ approach based on patient-specific fibrosis patterns, rather than a one-size-fits-all strategy, providing critical guidance for personalized optogenetic therapy.

### 4.6. The biophysical properties of the opsin as a determinant of control efficacy

Beyond illumination strategy and substrate heterogeneity, the intrinsic biophysical properties of the optogenetic actuator itself are a critical determinant of defibrillation efficacy. Our comprehensive sensitivity analysis demonstrates that while global uniform illumination successfully terminates spiral waves across the entire tested range of GtACR1 reversal potentials (−80 to −20 mV), the termination time is strongly modulated by this key parameter. The overall reduction in termination time with increasingly depolarized *E*_GtACR1_values is consistent with the generation of stronger depolarizing photocurrents, which effectively clamp a larger tissue mass into a refractory state, thereby accelerating wavefront annihilation. Strikingly, termination efficiency was lowest at *E*_GtACR1_= −60 mV, which lies close to the action potential threshold of human atrial myocytes. We hypothesize that at this critical potential, the photocurrent exerts a dual and opposing influence: it is insufficiently hyperpolarizing to stabilize resting potentials, yet too weakly depolarizing to induce sustained refractoriness. Instead, it may generate localized, subthreshold perturbations that transiently alter conduction velocity and wavelength, inadvertently increasing spatio-temporal complexity and delaying complete extinction. This non-monotonic response highlights a crucial design principle for therapeutic opsins: selection should prioritize not only expression efficiency and kinetics, but also a reversal potential sufficiently depolarized (e.g. greater than −50 mV) to ensure robust and rapid arrhythmia control. The excellent numerical convergence of this phenomenon across multiple spatio-temporal resolutions (supplementary material, tables S1 and S2) confirms its origin in genuine electrophysiological dynamics rather than computational artefacts.

### 4.7. The hierarchical determinants of optogenetic control robustness

The robustness of optogenetic spiral wave termination arises from a hierarchical interplay between substrate-determining microstructural features and modulatory macroscopic tissue properties. At the foundational level, the degree of local conduction slowing within fibrotic regions, which serves to model collagen-mediated gap junction disruption, emerges as the dominant determinant of rotor stability and intervention susceptibility. Enhanced uncoupling (e.g. 15%/45% conductivity reduction) strongly anchors spiral waves in focal fibrosis, markedly prolonging termination, whereas milder reductions facilitate faster annihilation. This sensitivity reflects the direct impact of microstructural topology on wavefront curvature and anchoring.

In stark contrast, global fibroblast-myocyte coupling conductance (*G*_gap_) exhibits no influence: perturbations from 1.0 to 3.0 nS leave termination times unchanged. This insensitivity stems from the dominant effect of subthreshold illumination, which clamps illuminated cardiomyocytes near *E*_GtACR1_, rendering them electrically silent regardless of fibroblast loading. Consequently, only the excitable tissue at the illumination boundary governs wave dynamics, where fibroblast coupling plays a negligible role.

Beyond conduction slowing and coupling conductance, random fluctuations in fibrosis spatial patterning also do not alter the core conclusions. The lower termination threshold of diffuse fibrosis, as well as the non-monotonic response of termination time to illumination coverage, remain highly consistent across multiple independent random configurations, with within-group variability far smaller than between-group differences driven by fibrosis percentage or illumination area. This indicates that the difference in control efficacy between the two fibrosis patterns is not determined by specific cellular arrangements, but by their overall spatial statistical properties—distributed conduction heterogeneity for diffuse fibrosis and rotor-anchoring effects for focal fibrosis. This property further strengthens the generalizability of our findings—the control principles revealed in this study remain valid even given the individual variability of fibrosis architecture in clinical patients.

At the spatial scale level, enlargement of the computational domain does not alter the core response patterns. The non-monotonic illumination response and the existence of an optimal window remain stable in the doubled domain, with fully overlapping optimal interval ranges and only a global increase in absolute termination time owing to extended wave propagation paths, indicating scale invariance of this dynamical rule that is not dominated by finite domain boundaries. In addition, the electrophysiological remodelling properties of fibrosis itself represent an important dimension of robustness. Recent studies indicate that fibrotic tissue is not simply a static conduction obstacle, but a dynamically remodelled substrate with frequency-dependent conduction abnormalities. Its conduction capacity declines with increasing stimulation rate, an effect rooted in the combined action of exacerbated source-sink mismatch, spatial redistribution of connexins, pathologically enhanced heterocellular electrical coupling and local ion channel remodelling. Our fibrosis model already incorporates ion channel mechanisms of cardiomyocytes and fibroblasts, as well as electrical coupling between the two cell types, at the cellular level to establish the cellular basis of conduction heterogeneity. Guided by the experimental findings of Giardini *et al.* [31], we introduced a rule of reduced fibrotic conductivity at high frequency into the 50% diffuse fibrosis model to verify the frequency robustness of our core rules. The results show that the optimal illumination window pattern remains entirely stable at low frequency. At high frequency, overall termination times are universally prolonged, and dynamical bifurcation occurs within a narrow interval around 70% illumination; some random realizations show elevated termination time owing to wavebreak and extra excitation waves, while others maintain a steady downward trend. This phenomenon is fully consistent with the low-pass filtering property of fibrotic tissue, suggesting that the optimal range of intermediate illumination coverage can be moderately narrowed to 40–60% at high frequencies to avoid the dynamically unstable region.

Similarly, variations in opsin biophysics—specifically the *E*_GtACR1_(from −80 to −20 mV)—modulate termination efficiency but do not abolish the core principle; full-field illumination invariably silences re-entry by suppressing global excitability. Numerical convergence tests further confirm that these phenomena are not artefacts of discretization; refinement of temporal and spatial resolution alters absolute times only marginally, preserving all qualitative trends.

Macroscopic tissue properties act as secondary modulators. Increasing conduction velocity to approximately 70 cm s^–1^ accelerates wavefront propagation, promoting earlier collisions and thus faster termination, yet the non-monotonic ‘optimal illumination range’ persists because it is governed by the relative geometry between the spiral core, illumination boundary and domain edge. This geometric relationship represents a topological invariant insensitive to absolute propagation speed. Likewise, introducing 2 : 1 structural anisotropy stabilizes rotors by promoting straighter wavefronts along the fast-conduction axis, significantly prolonging termination at intermediate illumination levels (e.g. 672 ms at 70% coverage in 75% fibrosis). Nevertheless, the fundamental response pattern endures: partial illumination can accelerate termination via wavefront collisions, excessive coverage suppresses such interactions, and complete illumination guarantees rapid annihilation.

Critically, across all parameter variations, molecular, cellular, microstructural and tissue-scale, the qualitative hierarchy remains intact; diffuse fibrosis is consistently more amenable to optogenetic control than focal fibrosis, and the non-monotonic dependence on illumination area is universally preserved. This multi-scale invariance underscores that the ‘optimal illumination range’ is not a model-specific artefact, but an emergent property of excitable media under spatially localized perturbation, rooted in the universal physics of nonlinear wave dynamics. Its physical origin lies in fundamental principles of nonlinear wave dynamics, including curvature-dependent wave propagation, core-boundary interactions and the intrinsic trade-off between excitable domain geometry and boundary annihilation efficiency. These principles are consistently observed across chemical oscillators, neural fields and cardiac tissue [40,41]. This universality carries profound clinical implications; even in the face of substantial inter-patient variability in fibrosis architecture, conduction velocity or tissue anisotropy, effective and low-energy rotor termination can be achieved by tailoring illumination to avoid both under- and over-coverage, guided primarily by fibrosis pattern recognition. Thus, the ‘optimal illumination range’ is more than a theoretical curiosity. It may serve as a foundational principle for personalized optical defibrillation strategies, where precise calibration of every biophysical parameter is unnecessary, and robust control instead emerges from respecting the underlying substrate topology. This insight provides a crucial theoretical bridge and practical roadmap for translating optogenetics from experimental platforms toward clinical atrial fibrillation therapy.

### 4.8. Limitations

This study systematically reveals the regulatory patterns and dynamical mechanisms of fibrosis spatial patterns on optogenetic spiral wave control via computational simulation. Constrained by the model framework and research scope, several limitations remain, which also clarify directions for future extension.

First, this study adopts a two-dimensional tissue model for mechanistic investigation, which cannot fully reproduce three-dimensional properties of the real atrium, including transmural electrophysiological heterogeneity, twist and break-up dynamics of scroll wave filaments and endocardial-epicardial electrical dissociation. Construction of a three-dimensional full-thickness atrial model with transmural heterogeneity to verify the applicability of our core findings in three-dimensional scenarios is a key research direction of our team.

Second, all simulations in this study are performed on a regular square computational domain. Although we have verified the robustness of the core dynamical rules by doubling the domain size and confirmed that boundary effects only modulate the absolute value of termination time without altering the dynamical nature of the phenomenon, regular geometric boundaries still differ notably from the irregular three-dimensional anatomy and complex chamber morphology of the real atrium, leading to quantitative differences in the spatial confinement effect. Follow-up studies may perform simulations on realistic atrial anatomical models reconstructed from clinical imaging to further improve the clinical relevance of the conclusions.

Third, this study only examines two idealized spatial patterns of fibrosis, diffuse and focal. In clinical practice, real atrial fibrosis often presents complex patient-specific spatial configurations such as patchy, reticular and border-zone distributions. Different fibrosis topologies may alter rotor anchoring properties and conduction heterogeneity, and thus, exert differential impacts on optogenetic termination efficacy. Follow-up studies may perform patient-specific modelling based on real fibrosis distributions extracted from clinical imaging to further improve the clinical relevance of conclusions.

Fourth, in this study, fibrosis-mediated electrophysiological remodelling is implemented mainly via phenomenological scaling of global conduction properties, and frequency-dependent conduction attenuation is also realized through proportional adjustment of bulk conductivity. The various remodelling factors related to fibrosis have not been independently parametrized at the molecular ion channel level. This approach can effectively capture macroscopic conduction rules, but cannot finely reflect the regulatory effects of channel-level remodelling on wave dynamics. Follow-up studies may construct multi-scale fibrosis models incorporating ion channel alterations to characterize conduction heterogeneity and frequency-dependent properties under pathological conditions more accurately.

Fifth, this study assumes uniform expression of GtACR1 across the entire tissue domain. In actual clinical scenarios, the delivery efficiency of viral vectors shows spatial variation, which may lead to heterogeneous distribution of opsin expression levels and even local regions with no optical response. The quantitative impacts of expression heterogeneity on optogenetic termination threshold and dynamical evolution require further systematic validation in future studies.

## 5. Conclusion

In summary, our computational modelling reveals three core principles governing the optogenetic control of spiral waves in fibrotic human atrial tissue. First, focal fibrosis exerts stronger rotor anchoring effects than diffuse fibrosis, so larger illuminated areas are required to achieve successful termination. Second, a robust optimal illumination range emerges across all tested substrates, in which intermediate coverage maximizes annihilation efficiency through a combination of geometric confinement and fragment wave front interactions, whereas both insufficient and excessive coverage impair overall intervention efficacy. Third, low-intensity periodic illumination can induce self-terminating spiral drift in diffuse fibrotic tissue, but this strategy fails in focal substrates owing to persistent structural anchoring of the rotor core. These insights were validated across a broad spectrum of biophysically plausible conditions, including variations in opsin biophysics, fibroblast coupling, conduction velocity and tissue anisotropy, confirming that the observed response patterns reflect inherent emergent properties of excitable media rather than model-specific idiosyncrasies. Collectively, these principles provide a robust, mechanism-informed foundation for the future development of personalized low-energy optical defibrillation strategies tailored to individual fibrosis phenotypes.

## Supplementary Material



## Data Availability

The baseline human atrial myocyte electrophysiology model was originally developed by Courtemanche *et al.*, and its standard CellML implementation is publicly available in the CellML Model Repository: https://models.cellml.org/exposure/0e03bbe01606be5811691f9d5de10b65. The myocyte-fibroblast electrotonic coupling model follows the formulation established by Maleckar *et al.*, with its CellML version accessible at: https://models.cellml.org/e/806. The GtACR1 optogenetic current model, originally developed by Boyle *et al.*, is publicly available via figshare [42]. Its two-state kinetic framework and full parameter settings are also restated in detail in §2.3 of this manuscript. Two-dimensional tissue-scale simulations of spiral wave dynamics were performed using a custom Fortran-based solver. To facilitate independent reproduction of our core findings, the methods section provides a thorough description of all necessary model components, including cellular model equations, fibrosis representation protocols (diffuse and focal patterns), illumination stimulation schemes and complete parameter sets. These components can be reconstructed within the open-source cardiac electrophysiology simulation platform openCARP (https://www.opencarp.org) to replicate the spiral wave dynamics and termination outcomes reported in this work. Supplementary material is available online [43].

## References

[1] Rappel W-J , Krummen DE, Baykaner T, Zaman J, Donsky A, Swarup V, Miller JM, Narayan SM. 2022 Stochastic termination of spiral wave dynamics in cardiac tissue. Front. Netw. Physiol. 2, 809532. (doi:10.3389/fnetp.2022.809532) PMC9524168 36187938

[2] Majumder R . 2024 In silico thermal control of spiral wave dynamics in excitable cardiac tissue. Biophys. Rep. 4, 100170. (doi:10.1016/j.bpr.2024.100170) PMC11304022 38960373

[3] Pang Z , Ren Y, Yao Z. 2025 Interactions between atrial fibrosis and inflammation in atrial fibrillation. Front. Cardiovasc. Med. 12, 1578148. (doi:10.3389/fcvm.2025.1578148) 40709209 PMC12287038

[4] Hermans BJM , Weberndörfer V, Bijvoet GP, Chaldoupi S-M, Linz D. 2022 New concepts in atrial fibrillation pathophysiology. Herzschrittmacherther Elektrophysiol. 33, 362–366. (doi:10.1007/s00399-022-00897-1) 36136132 PMC9691491

[5] Zimik S , Pandit R, Majumder R. 2020 Anisotropic shortening in the wavelength of electrical waves promotes onset of electrical turbulence in cardiac tissue: an* in silico* study. PLoS ONE 15, e0230214. (doi:10.1371/journal.pone.0230214) 32168323 PMC7069633

[6] Hussaini S *et al.* 2024 Efficient termination of cardiac arrhythmias using optogenetic resonant feedback pacing. Chaos 34, 031103. (doi:10.1063/5.0191519) 38526981

[7] Suth D , Luther S, Lilienkamp T. 2024 Chaos control in cardiac dynamics: terminating chaotic states with local minima pacing. Front. Netw. Physiol. 4, 1401661. (doi:10.3389/fnetp.2024.1401661) 39022296 PMC11252590

[8] Boyle PM , Karathanos TV, Entcheva E, Trayanova NA. 2015 Computational modeling of cardiac optogenetics: methodology overview & review of findings from simulations. Comput. Biol. Med. 65, 200–208. (doi:10.1016/j.compbiomed.2015.04.036) 26002074 PMC4591100

[9] Leemann S , Schneider-Warme F, Kleinlogel S. 2023 Cardiac optogenetics: shining light on signaling pathways. Pflug. Arch. 475, 1421–1437. (doi:10.1007/s00424-023-02892-y) PMC10730638 38097805

[10] Karakasis P , Theofilis P, Vlachakis PK, Milaras N, Kalinderi K, Patoulias D, Antoniadis AP, Fragakis N. 2025 Gene therapy for cardiac arrhythmias: mechanisms, modalities and therapeutic applications. Med. Sci. 13, 102. (doi:10.3390/medsci13030102) PMC12372054 40843724

[11] Govorunova EG , Sineshchekov OA, Spudich JL. 2021 Emerging diversity of channelrhodopsins and their structure-function relationships. Front. Cell Neurosci. 15, 800313. (doi:10.3389/fncel.2021.800313) 35140589 PMC8818676

[12] Chua CJ , Han JL, Li W, Liu W, Entcheva E. 2021 Integration of engineered “spark-cell” spheroids for optical pacing of cardiac tissue. Front. Bioeng. Biotechnol. 9, 658594. (doi:10.3389/fbioe.2021.658594) 34222210 PMC8249938

[13] McDowell KS , Vadakkumpadan F, Blake R, Blauer J, Plank G, MacLeod RS, Trayanova NA. 2012 Methodology for patient-specific modeling of atrial fibrosis as a substrate for atrial fibrillation. J. Electrocardiol. 45, 640–645. (doi:10.1016/j.jelectrocard.2012.08.005) 22999492 PMC3515859

[14] Biasci V *et al.* 2022 Optogenetic manipulation of cardiac electrical dynamics using sub-threshold illumination: dissecting the role of cardiac alternans in terminating rapid rhythms. Basic Res. Cardiol. 117, 25. (doi:10.1007/s00395-022-00933-8) 35488105 PMC9054908

[15] Plank G *et al.* 2021 The openCARP simulation environment for cardiac electrophysiology. Comput. Methods Programs Biomed. 208, 106223. (doi:10.1016/j.cmpb.2021.106223) 34171774

[16] Entcheva E , Kay MW. 2021 Cardiac optogenetics: a decade of enlightenment. Nat. Rev. Cardiol. 18, 349–367. (doi:10.1038/s41569-020-00478-0) 33340010 PMC8127952

[17] Boyle PM *et al.* 2019 Computationally guided personalized targeted ablation of persistent atrial fibrillation. Nat. Biomed. Eng. 3, 870–879. (doi:10.1038/s41551-019-0437-9) 31427780 PMC6842421

[18] Luo J , Lu J, Yan H, Li Q, Panfilov AV, Li T-C. 2024 Unpinning and elimination of spiral waves and turbulence through local optogenetical illumination. Phys. Rev. E 110, 024218. (doi:10.1103/PhysRevE.110.024218) 39295036

[19] Aryana A *et al.* 2021 Left atrial posterior wall isolation in conjunction with pulmonary vein isolation using cryoballoon for treatment of persistent atrial fibrillation (PIVoTAL): study rationale and design. J. Interv. Card. Electrophysiol. 62, 187–198. (doi:10.1007/s10840-020-00885-w) 33009645 PMC8210744

[20] Masè M , Cristoforetti A, Pelloni S, Ravelli F. 2024 Systematic in-silico evaluation of fibrosis effects on re-entrant wave dynamics in atrial tissue. Sci. Rep. 14, 11427. (doi:10.1038/s41598-024-62002-5) 38763959 PMC11639732

[21] Marchal GA , Biasci V, Loew LM, Biggeri A, Campione M, Sacconi L. 2023 Optogenetic manipulation of cardiac repolarization gradients using sub-threshold illumination. Front. Physiol. 14, 1167524. (doi:10.3389/fphys.2023.1167524) 37215182 PMC10196067

[22] Courtemanche M , Ramirez RJ, Nattel S. 1998 Ionic mechanisms underlying human atrial action potential properties: insights from a mathematical model. Am. J. Physiol. 275, H301–H321. (doi:10.1152/ajpheart.1998.275.1.H301) 9688927

[23] Maleckar MM , Greenstein JL, Giles WR, Trayanova NA. 2009 Electrotonic coupling between human atrial myocytes and fibroblasts alters myocyte excitability and repolarization. Biophys. J. 97, 2179–2190. (doi:10.1016/j.bpj.2009.07.054) 19843450 PMC2764083

[24] Ochs AR , Karathanos TV, Trayanova NA, Boyle PM. 2021 Optogenetic stimulation using anion channelrhodopsin (GtACR1) facilitates termination of reentrant arrhythmias with low light energy requirements: a computational study. Front. Physiol. 12, 718622. (doi:10.3389/fphys.2021.718622) 34526912 PMC8435849

[25] Govorunova EG , Cunha SR, Sineshchekov OA, Spudich JL. 2016 Anion channelrhodopsins for inhibitory cardiac optogenetics. Sci. Rep. 6, 33530. (doi:10.1038/srep33530) 27628215 PMC5024162

[26] Colli Franzone P , Pavarino LF, Taccardi B. 2005 Simulating patterns of excitation, repolarization and action potential duration with cardiac bidomain and monodomain models. Math. Biosci. 197, 35–66. (doi:10.1016/j.mbs.2005.04.003) 16009380

[27] Hussaini S , Lädke SL, Schröder-Schetelig J, Venkatesan V, Quiñonez Uribe RA, Richter C, Majumder R, Luther S. 2023 Dissolution of spiral wave's core using cardiac optogenetics. PLoS Comput. Biol. 19, e1011660. (doi:10.1371/journal.pcbi.1011660) 38060618 PMC10729946

[28] Kostecki GM , Shi Y, Chen CS, Reich DH, Entcheva E, Tung L. 2021 Optogenetic current in myofibroblasts acutely alters electrophysiology and conduction of co-cultured cardiomyocytes. Sci. Rep. 11, 4430. (doi:10.1038/s41598-021-83398-4) 33627695 PMC7904933

[29] MacCannell KA , Bazzazi H, Chilton L, Shibukawa Y, Clark RB, Giles WR. 2007 A mathematical model of electrotonic interactions between ventricular myocytes and fibroblasts. Biophys. J. 92, 4121–4132. (doi:10.1529/biophysj.106.101410) 17307821 PMC1868994

[30] Macheret F , Bifulco SF, Scott GD, Kwan KT, Chahine Y, Afroze T, McDonagh R, Akoum N, Boyle PM. 2023 Comparing inducibility of re-entrant arrhythmia in patient-specific computational models to clinical atrial fibrillation phenotypes. JACC Clin. Electrophysiol. 9, 2149–2162. (doi:10.1016/j.jacep.2023.06.015) 37656099 PMC10909381

[31] Giardini F *et al.* 2025 Correlative imaging integrates electrophysiology with three-dimensional murine heart reconstruction to reveal electrical coupling between cell types. Nat. Cardiovasc. Res. 4, 1466–1486. (doi:10.1038/s44161-025-00728-9) 41053445 PMC12611784

[32] DeTal N , Kaboudian A, Fenton FH. 2022 Terminating spiral waves with a single designed stimulus: teleportation as the mechanism for defibrillation. Proc. Natl Acad. Sci. USA 119, e2117568119. (doi:10.1073/pnas.2117568119) 35679346 PMC9214532

[33] Li Q-H , Xia Y-X, Xu S-X, Song Z, Pan J-T, Panfilov AV, Zhang H. 2022 Control of spiral waves in optogenetically modified cardiac tissue by periodic optical stimulation. Phys. Rev. E 105, 044210. (doi:10.1103/PhysRevE.105.044210) 35590553

[34] Roney CH *et al.* 2020 *In silico* comparison of left atrial ablation techniques that target the anatomical, structural, and electrical substrates of atrial fibrillation. Front. Physiol. 11, 1145. (doi:10.3389/fphys.2020.572874) 33041850 PMC7526475

[35] Xia YX , Xie LH, He YJ, Pan JT, Panfilov AV, Zhang H. 2023 Numerical study of the drift of scroll waves by optical feedback in cardiac tissue. Phys. Rev. E 108, 064406. (doi:10.1103/PhysRevE.108.064406) 38243456

[36] Nizamieva AA , Kalita IY, Slotvitsky MM, Berezhnoy AK, Shubina NS, Frolova SR, Tsvelaya VA, Agladze KI. 2023 Conduction of excitation waves and reentry drift on cardiac tissue with simulated photocontrol-varied excitability. Chaos 33, 023112. (doi:10.1063/5.0122273) 36859193

[37] Hussaini S *et al.* 2021 Drift and termination of spiral waves in optogenetically modified cardiac tissue at sub-threshold illumination. eLife 10, e59954. (doi:10.7554/eLife.59954) 33502313 PMC7840178

[38] Hussaini S , Majumder R, Krinski V, Luther S. 2023 In silico optical modulation of spiral wave trajectories in cardiac tissue. Pflug. Arch. 475, 1453–1461. (doi:10.1007/s00424-023-02889-7) PMC10730633 38095694

[39] Bruegmann T , Beiert T, Vogt CC, Schrickel JW, Sasse P. 2018 Optogenetic termination of atrial fibrillation in mice. Cardiovasc. Res. 114, 713–723. (doi:10.1093/cvr/cvx250) 29293898

[40] Byrne Á , Ross J, Nicks R, Coombes S. 2022 Mean-field models for EEG/MEG: from oscillations to waves. Brain Topogr. 35, 36–53. (doi:10.1007/s10548-021-00842-4) 33993357 PMC8813727

[41] Tompkins N , Li N, Girabawe C, Heymann M, Ermentrout GB, Epstein IR, Fraden S. 2014 Testing Turing's theory of morphogenesis in chemical cells. Proc. Natl Acad. Sci. USA 111, 4397–4402. (doi:10.1073/pnas.1322005111) 24616508 PMC3970514

[42] Boyle PM , Ochs AR. 2021 Computational Simulation of Optogenetic Stimulation via ChR2, ChR2-RED, or GtACR1 in patient-derived ventricular models - Example [Dataset]. Figshare. (doi:10.6084/m9.figshare.14945412)

[43] Zhan H , Liu K, Ju Y, Wei C, Deng D, Yu Y. 2026 Supplementary material from: Optimal control of spiral waves in heterogeneous excitable media: non-monotonic efficacy of localized optogenetic inhibition. Figshare. (doi:10.6084/m9.figshare.c.8605227) PMC13561037 42712242

