# Supplementary material for: Optimal control of spiral waves in heterogeneous excitable media: non-monotonic efficacy of localized optogenetic inhibition
Source: J R Soc Interface. 2026 Sep 9;23(242):20251341. doi: 10.1098/rsif.2025.1341 (PMC13561037; doi:10.1098/rsif.2025.1341)
Supplement: Supplementary file 1 [file rsif.2025.1341.zip › rsif.2025.1341/rsif20251341_si_001.docx]

**Fig. S1.** Overall trends of spiral wave termination time across stochastic fibrosis realizations. Left: diffuse fibrosis; right: focal fibrosis. Colored curves correspond to four fibrosis coverage levels: 25%, 50%, 75%, and 100%. Lines represent mean termination time, with standard deviation shown as shaded bands for diffuse fibrosis and error bars for focal fibrosis. No standard deviation is presented for 100% fibrosis, as the fully fibrotic domain adopts a uniform configuration with no independent stochastic realizations.

**Fig. S2.** Distribution of individual replicate termination times across experimental conditions. Top row: diffuse fibrosis; bottom row: focal fibrosis. Columns correspond to 25%, 50%, 75%, and 100% fibrosis coverage from left to right. Black lines with markers show mean termination time; error bars show standard deviation; grey dots indicate results from individual independent stochastic realizations. Each condition includes 10 independent replicates for diffuse fibrosis and 3 for focal fibrosis. No individual replicates or error bars are presented for 100% fibrosis due to its uniform spatial structure.

**Fig. S3.** Comparison of termination time trends across different computational domain sizes. Blue line: enlarged 5.0×5.0 cm^2^ domain; orange line: original 2.5×2.5 cm^2^ domain. Both datasets are derived from 50% diffuse fibrosis substrates. The light grey shaded band marks the shared optimal illumination window range for both domains, concentrated at 40%–80% illumination coverage. All data points represent means across 10 independent random realizations, with error bars indicating standard deviation.

**Fig. S4.** Comparison of termination time trends across different stimulation frequencies. Orange line: 5 Hz low-frequency pacing; blue line: 10 Hz high-frequency pacing. The light grey shaded band marks the general optimal illumination window range, and the light red shaded band indicates the dynamically unstable interval at high frequency. All data points represent means across 10 independent random realizations, with error bars indicating standard deviation.

**Table S1.** Spiral wave termination times (ms) under global illumination (0.1 $\text{μ}$W/mm^2^) for varying GtACR1 reversal potentials ($\text{E}_{\text{GtACR1}}$) and numerical resolutions in 50% focal fibrosis.

| $\text{E}_{\text{GtACR1}}$ | $\text{−}$80 mV | $\text{−}$60 mV | $\text{−}$50 mV | $\text{−}$40 mV | $\text{−}$30 mV | $\text{−}$20 mV |
| --- | --- | --- | --- | --- | --- | --- |
| $\text{dx}\text{ }\text{=}\text{ }\text{0.025}\text{ }\text{cm}$  $\text{dt}\text{ = 10 }\text{μs}$ | 38 ms | 50 ms | 50 ms | 40 ms | 38 ms | 34 ms |
| $\text{dx}/{\sqrt{\text{2}}}\text{=}\text{ }\text{0.0}\text{177 }\text{cm}$  $\text{dt}\text{ = 10 }\text{μs}$ | 40 ms | 64 ms | 50 ms | 50 ms | 36 ms | 34 ms |
| $\text{dx}\text{=0.025}\text{ }\text{cm}$  $\text{dt}\text{/2 }\text{= 5 }\text{μs}$ | 38 ms | 50 ms | 50 ms | 40 ms | 38 ms | 34 ms |
| $\text{dx}/{\sqrt{\text{2}}}\text{=}\text{ }\text{0.0}\text{177 }\text{cm}$  $\text{dt}\text{/2 }\text{= 5 }\text{μs}$ | 40 ms | 64 ms | 50 ms | 50 ms | 36 ms | 34 ms |

**Table S2.** Spiral wave termination times (ms) under global illumination (0.1 $\text{μ}$W/mm^2^) for varying $\text{E}_{\text{GtACR1}}$ and numerical resolutions in 50% diffuse fibrosis.

| $\text{E}_{\text{GtACR1}}$ | $\text{−}$80 mV | $\text{−}$60 mV | $\text{−}$50 mV | $\text{−}$40 mV | $\text{−}$30 mV | $\text{−}$20 mV |
| --- | --- | --- | --- | --- | --- | --- |
| $\text{dx}\text{ }\text{=}\text{ }\text{0.025}\text{ }\text{cm}$  $\text{dt}\text{ = 10 }\text{μs}$ | 40 ms | 62 ms | 52 ms | 40 ms | 36ms | 32 ms |
| $\text{dx}/{\sqrt{\text{2}}}\text{=}\text{ }\text{0.0}\text{177 }\text{cm}$  $\text{dt}\text{ = 10 }\text{μs}$ | 50 ms | 54 ms | 50 ms | 38 ms | 34 ms | 30 ms |
| $\text{dx}\text{=0.025}\text{ }\text{cm}$  $\text{dt}\text{/2 }\text{= 5 }\text{μs}$ | 40 ms | 62 ms | 52ms | 40 ms | 36 ms | 32 ms |
| $\text{dx}/{\sqrt{\text{2}}}\text{=}\text{ }\text{0.0}\text{177 }\text{cm}$  $\text{dt}\text{/2 }\text{= 5 }\text{μs}$ | 50 ms | 54 ms | 50 ms | 38 ms | 34 ms | 30 ms |

**Table S3.** Spiral wave termination times (ms) under varying illumination coverage in 50% focal fibrosis, for different assumptions of gap junction conductance ($\text{G}_{\text{gap}}$) and fibrosis-induced intercellular conductance reductions.

|  |  | Illumination coverage (%) | | | | | |
| --- | --- | --- | --- | --- | --- | --- | --- |
| $\text{G}_{\text{gap}}\text{↓}$ (%) | $\text{G}_{\text{gap}}$ (nS) | 50% | 60% | 70% | 80% | 90% | 100% |
| 5% and 15% | 1.0 | 232ms | 178ms | 178ms | 178ms | 176ms | 40ms |
| 5% and 15% | 1.5 | 232ms | 178ms | 178ms | 178ms | 176ms | 40ms |
| 5% and 15% | 2.0 | 232ms | 178ms | 178ms | 178ms | 176ms | 40ms |
| 5% and 15% | 2.5 | 232ms | 178ms | 178ms | 178ms | 176ms | 40ms |
| 5% and 15% | 3.0 | 232ms | 178ms | 178ms | 178ms | 176ms | 40ms |
| 10% and 30% | 1.0 | 236ms | 236ms | 232ms | 264ms | 230ms | 40ms |
| 10% and 30% | 1.5 | 236ms | 236ms | 232ms | 264ms | 230ms | 40ms |
| 10% and 30% | 2.0 | 236ms | 236ms | 232ms | 264ms | 230ms | 40ms |
| 10% and 30% | 2.5 | 236ms | 236ms | 232ms | 264ms | 230ms | 40ms |
| 10% and 30% | 3.0 | 236ms | 236ms | 232ms | 264ms | 230ms | 40ms |
| 15% and 45% | 1.0 | 590ms | 748ms | 748ms | 328ms | 326ms | 40ms |
| 15% and 45% | 1.5 | 590ms | 748ms | 748ms | 328ms | 326ms | 40ms |
| 15% and 45% | 2.0 | 590ms | 748ms | 748ms | 328ms | 326ms | 40ms |
| 15% and 45% | 2.5 | 590ms | 748ms | 748ms | 328ms | 326ms | 40ms |
| 15% and 45% | 3.0 | 590ms | 748ms | 748ms | 328ms | 326ms | 40ms |

**Table S4.** Spiral wave termination times (ms) under varying illumination coverage in 50% diffuse fibrosis, for different assumptions of $\text{G}_{\text{gap}}$and fibrosis-induced intercellular conductance reductions.

|  |  | Illumination coverage (%) | | | | | |
| --- | --- | --- | --- | --- | --- | --- | --- |
| $\text{G}_{\text{gap}}\text{↓}$ (%) | $\text{G}_{\text{gap}}$ (nS) | 50% | 60% | 70% | 80% | 90% | 100% |
| 5% and 15% | 1.0 | 214ms | 214ms | 214ms | 214ms | 234ms | 40ms |
| 5% and 15% | 1.5 | 214ms | 214ms | 214ms | 214ms | 234ms | 40ms |
| 5% and 15% | 2.0 | 214ms | 214ms | 214ms | 214ms | 234ms | 40ms |
| 5% and 15% | 2.5 | 214ms | 214ms | 214ms | 214ms | 234ms | 40ms |
| 5% and 15% | 3.0 | 214ms | 214ms | 214ms | 214ms | 234ms | 40ms |
| 10% and 30% | 1.0 | 240ms | 240ms | 236ms | 236ms | 322ms | 40ms |
| 10% and 30% | 1.5 | 240ms | 240ms | 236ms | 236ms | 322ms | 40ms |
| 10% and 30% | 2.0 | 240ms | 240ms | 236ms | 236ms | 322ms | 40ms |
| 10% and 30% | 2.5 | 240ms | 240ms | 236ms | 236ms | 322ms | 40ms |
| 10% and 30% | 3.0 | 240ms | 240ms | 236ms | 236ms | 322ms | 40ms |
| 15% and 45% | 1.0 | 242ms | 242ms | 242ms | 304ms | 40ms | 40ms |
| 15% and 45% | 1.5 | 242ms | 242ms | 242ms | 304ms | 40ms | 40ms |
| 15% and 45% | 2.0 | 242ms | 242ms | 242ms | 304ms | 40ms | 40ms |
| 15% and 45% | 2.5 | 242ms | 242ms | 242ms | 304ms | 40ms | 40ms |
| 15% and 45% | 3.0 | 242ms | 242ms | 242ms | 304ms | 40ms | 40ms |

**Table S5.** Spiral wave termination times (ms) in 50% diffuse fibrosis under baseline (CV=43.9cm/s) and clinically representative conduction velocity (CV=70cm/s), across illumination coverage from 50% to 100%.

| Illumination coverage (%) | 50% | 60% | 70% | 80% | 90% | 100% |
| --- | --- | --- | --- | --- | --- | --- |
| CV = 70 cm/s | 104ms | 104ms | 102ms | 178ms | 46ms | 38ms |
| CV = 43.9 cm/s | 240ms | 240ms | 236ms | 236ms | 322ms | 40ms |

**Table S6.** Spiral wave termination times (ms) in diffuse fibrosis (50%, 75%, and 100%) under isotropic (dx=dy) and anisotropic (dx=2dy) tissue models, across illumination coverage from 50% to 100%.

| Illumination coverage (%) | | 50% | 60% | 70% | 80% | 90% | 100% |
| --- | --- | --- | --- | --- | --- | --- | --- |
| 50% diffuse fibrosis | dx = 2dy | 356ms | 356ms | 356ms | 582ms | 196ms | 38ms |
|  | dx = dy | 240ms | 240ms | 236ms | 236ms | 322ms | 40ms |
| 75% diffuse fibrosis | dx = 2dy | 482ms | 480ms | 672ms | 220ms | 218ms | 38ms |
|  | dx = dy | 276ms | 276ms | 346ms | 142ms | 138ms | 40ms |
| 100% diffuse fibrosis | dx = 2dy | 384ms | 384ms | 610ms | 244ms | 242ms | 38ms |
|  | dx = dy | 240ms | 232ms | 232ms | 286ms | 38ms | 38ms |

**Table S7.** Mean±standard deviation of spiral wave termination time (ms) across stochastic fibrosis realizations.

(A) Diffuse fibrosis

| Illuminated area (%) | 25% Fibrosis | 50% Fibrosis | 75% Fibrosis | 100% Fibrosis |
| --- | --- | --- | --- | --- |
| 10% | — | — | — | — |
| 20% | 570.2±11.17 | — | 286.2±6.96 | — |
| 30% | 364.6±11.66 | 429.4±10.16 | 284.4±8.21 | 572.0* |
| 40% | 363.4±11.70 | 234.4±9.47 | 282.6±6.80 | 240.0* |
| 50% | 163.0±11.05 | 233.8±9.82 | 282.6±6.80 | 240.0* |
| 60% | 161.5±10.97 | 232.6±9.80 | 281.2±7.19 | 232.0* |
| 70% | 160.6±10.79 | 229.6±9.51 | 352.4±6.85 | 232.0* |
| 80% | 159.8±10.60 | 228.0±9.04 | 141.8±6.36 | 286.0* |
| 90% | 161.0±9.01 | 333.6±9.70 | 141.2±6.12 | 38.0* |
| 100% | 38.2±1.48 | 38.8±1.03 | 39.0±1.41 | 38.0* |

(B) Focal fibrosis

| Illuminated area (%) | 25% Fibrosis | 50% Fibrosis | 75% Fibrosis | 100% Fibrosis |
| --- | --- | --- | --- | --- |
| 10% | — | — | — | — |
| 20% | — | — | — | — |
| 30% | 1075.33±1.15 | 1120.00±0 | 796.00±0 | 572.0* |
| 40% | 734.67±2.31 | 434.00±0 | 282.00±0 | 240.0* |
| 50% | 200.00±0 | 236.00±0 | 278.00±0 | 240.0* |
| 60% | 200.00±0 | 236.00±0 | 272.00±0 | 232.0* |
| 70% | 199.33±1.15 | 232.67±1.15 | 272.00±0 | 232.0* |
| 80% | 192.00±0 | 264.00±0 | 292.00±0 | 286.0* |
| 90% | 190.00±0 | 230.00±0 | 286.00±0 | 38.0* |
| 100% | 36.67±1.15 | 40.00±0 | 36.00±0 | 38.0* |

Note: “—” indicates no successful termination within 3000 ms. For 100% fibrosis, the spatial configuration is uniform across the entire domain with no independent stochastic realizations, hence no standard deviation is reported. Sample size: n = 10 per condition for non-uniform diffuse fibrosis; n = 3 per condition for non-uniform focal fibrosis.

**Table S8.** Mean±standard deviation of termination time (ms) for 50% diffuse fibrosis across different computational domain sizes.

| Illuminated area (%) | 2.5×2.5 cm^2^ domain | 5.0×5.0 cm^2^ domain |
| --- | --- | --- |
| 10% | — | — |
| 20% | — | — |
| 30% | 429.4±10.16 | 587.8±20.96 |
| 40% | 234.4±9.47 | 421.2±16.50 |
| 50% | 233.8±9.82 | 418.8±15.64 |
| 60% | 232.6±9.80 | 418.8±15.64 |
| 70% | 229.6±9.51 | 417.2±17.31 |
| 80% | 228.0±9.04 | 414.2±16.80 |
| 90% | 333.6±9.70 | 478.0±16.19 |
| 100% | 38.8±1.03 | 38.0±1.63 |

Note: “—” indicates no successful termination within 3000 ms. n = 10 per condition.

**Table S9.** Mean±standard deviation of termination time (ms) for 50% diffuse fibrosis under different stimulation frequencies.

| Illuminated area (%) | 5 Hz Low frequency | 10 Hz High frequency |
| --- | --- | --- |
| 10% | — | — |
| 20% | — | — |
| 30% | 429.4±10.16 | 569.8±12.56 |
| 40% | 234.4±9.47 | 362.6±11.89 |
| 50% | 233.8±9.82 | 361.6±12.36 |
| 60% | 232.6±9.80 | 362.6±8.49 |
| 70% | 229.6±9.51 | 314.0±101.19 |
| 80% | 228.0±9.04 | 158.2±11.25 |
| 90% | 333.6±9.70 | 156.6±10.88 |
| 100% | 38.8±1.03 | 39.4±0.97 |

Note: “—” indicates no successful termination within 3000 ms. n = 10 per condition. The markedly elevated standard deviation at 10 Hz and 70% illumination arises from dynamical bifurcation induced by extra excitation waves in a subset of random realizations.
